# A systematic review of proxy-report questionnaires assessing physical activity, sedentary behavior and/or sleep in young children (aged 0–5 years)

**DOI:** 10.1186/s12966-022-01251-x

**Published:** 2022-02-14

**Authors:** Jelle Arts, Jessica S. Gubbels, Arnoud P. Verhoeff, Mai. J. M. Chinapaw, Annelinde Lettink, Teatske M. Altenburg

**Affiliations:** 1 Department of Public and Occupational Health, Amsterdam UMC, Vrije Universiteit Amsterdam, Amsterdam Public Health research institute, Van der Boechorststraat 7, 1081 BT Amsterdam, The Netherlands; 2 Department of Health Promotion, NUTRIM School of Nutrition and Translational Research in Metabolism, Maastricht University, PO Box 616, 6200 MD Maastricht, The Netherlands; 3 Public Health Service Amsterdam, Sarphati Amsterdam, 1018 WT Amsterdam, The Netherlands; 4 Department of Sociology, University of Amsterdam, 1018 WV Amsterdam, The Netherlands

**Keywords:** 24-h movement behaviors, Infants, Toddlers, Preschoolers, Questionnaires, Parent-report, Measurement properties, Validity, Reliability

## Abstract

**Background:**

Accurate proxy-report questionnaires, adapted to the child’s developmental stage, are required to monitor 24-h movement behaviors in young children, especially for large samples and low-resource settings.

**Objectives:**

This review aimed to summarize available studies evaluating measurement properties of proxy-report questionnaires assessing physical activity, sedentary behavior and/or sleep in children aged 0–5 years.

**Methods:**

Systematic literature searches were carried out in the PubMed, Embase and SPORTDiscus databases, up to January 2021. For physical activity and sedentary behavior questionnaires this is a review update, whereas for sleep questionnaires we included all relevant studies published up to now. Studies had to evaluate at least one of the measurement properties of a proxy-report questionnaire assessing at least duration and/or frequency of physical activity, sedentary behavior and/or sleep in 0- to 5-year-old children. The COnsensus-based Standards for the selection of health Measurement INstruments (COSMIN) guideline was used to evaluate the quality of evidence.

**Results:**

Thirty-three studies were included, examining a total of 37 questionnaires. Ten questionnaires were designed for infants, two for toddlers, 11 for preschoolers, and 14 for a broader age range targeting multiple of these age groups. Twenty questionnaires assessed constructs of sleep, four assessed constructs of physical activity, two assessed screen behavior, five assessed constructs of both physical activity and sedentary behavior, and six assessed constructs of all 24-h movement behaviors. Content validity was evaluated for six questionnaires, structural validity for two, internal consistency for three, test-retest reliability for 16, measurement error for one, criterion validity for one, and construct validity for 26 questionnaires. None of the questionnaires were considered sufficiently valid and/or reliable for assessing one or more movement behaviors in 0- to 5-year-old children, and the quality of evidence was mostly low or very low.

**Conclusions:**

Valid and/or reliable questionnaires assessing 24-h movement behaviors in 0- to 5-year-olds are lacking. High-quality studies are therefore required, to develop proxy-report questionnaires and evaluate their measurement properties.

**PROSPERO registration number:**

CRD42020169268.

**Supplementary Information:**

The online version contains supplementary material available at 10.1186/s12966-022-01251-x.

## Background

Establishing healthy movement behaviors in early childhood is necessary to support the growth and development of young children and the maintenance of their long-term health [1–4]. Recent studies indicate the importance of the combination of all 24-h movement behaviors, encompassing physical activity, sedentary behavior, and sleep [5–10]. Valid, reliable, responsive, affordable, and feasible measurement instruments adapted to the child’s developmental stage are required to monitor 24-h movement behaviors in young children. Direct observation is considered a suitable criterion measure of different movement behaviors in children [11, 12]. However, observation can be very time consuming and thus costly, and is thereby not feasible to use on a large scale. Alternatively, physical activity, sedentary behavior and sleep in young children can be measured using accelerometers [3, 12, 13]. Although accelerometers are considered valid and reliable for measuring movement behaviors in children and adolescents [14, 15], its validity is yet to be obtained for the youngest age group (i.e., infants: 0–1 year old) [13]. In addition, accelerometer output is processed by a number of subjective decisions in order to translate acceleration data into time estimates of movement behaviors. Specifically, it is unknown which analyses methods provide the most accurate classification of physical activity, sedentary behavior and sleep in infants. Additionally, current procedures do not take into account that accelerometer output in very young children may reflect movements of others, e.g., that of parents who carry their child or push them in a stroller [16]. Alternative to observation and accelerometry, parent- or caregiver (proxy-) report questionnaires can be used as a measurement instrument for children’s 24-h movement behaviors. Questionnaires can be used to assess movement behaviors on a large scale in a relatively convenient and affordable way, with the additional advantage of obtaining information about the type (e.g., active play, screen time) and context (e.g., outside, alone) of the behavior. Unfortunately, proxy-report questionnaires have their own limitations such as recall and social desirability bias [17]. Furthermore, the intermittent and unstructured pattern of movement behaviors in young children complicates accurate reporting of these behaviors [17].

To date, a number of proxy-report questionnaires have been developed to assess physical activity, sedentary behavior and/or sleep in 0- to 5-year-old children. A few systematic reviews examined the measurement properties of these questionnaires in young children [18–21], searching the literature up to 2014 for questionnaires on sleep [18], up to 2015 for sedentary behavior [20] and up to 2018 for physical activity [19, 21]. However, concerning questionnaires on sleep, questionnaires for the youngest age group were not evaluated (i.e., 0- to 2-year-old children) [18]. Unfortunately, these reviews did not identify any questionnaires that can be considered both reliable and valid for assessing physical activity, sedentary behavior and/or sleep in children aged < 5 years [18–21]. Although a review published in 2011 [22] and updated in 2020 [23] provided an overview of the psychometric analyses performed at the available pediatric sleep questionnaires, including questionnaires for 0- to 5-year-old children, measurement property results were not reported in the review update [23]. In addition, quality of evidence was not considered in both reviews, limiting the conclusions that can be drawn regarding the best available questionnaires [22, 23]. Similarly, a recently published systematic review provided an overview of measurement tools used to assess screen time in 0- to 6-year-old children [22]. Although measurement properties of the available questionnaires were reported, a comprehensive evaluation of the measurement properties, including quality of evidence, was lacking [24]. Furthermore, as the research interest of all 24-h movement behaviors in young children has grown, new questionnaires might have been developed over the last few years. For this reason, an update of previous reviews is required, including physical activity, sedentary behavior and sleep. To be able to select the most appropriate questionnaires to assess 24-h movement behaviors, an overview of the characteristics and measurement properties of the available questionnaires for young children is highly warranted.

Therefore, the purpose of this review was to summarize all studies examining measurement properties (e.g., reliability and validity) of proxy-report questionnaires assessing physical activity, sedentary behavior and/or sleep in children aged 0–5 years. We evaluated the quality of evidence for each measurement property, including a methodological quality assessment of included studies. Additionally, we provided an overview of the characteristics of the evaluated questionnaires (e.g., the target population and format of the questionnaires).

## Methods

We registered this review on PROSPERO (international prospective register of systematic reviews; registration number: CRD42020169268) and followed the Preferred Reporting Items for Systematic Reviews and Meta-Analyses (PRISMA) guidelines [25].

### Literature search

Systematic literature searches were carried out in PubMed, Embase, and SPORTDiscus. For physical activity and sedentary behavior questionnaires this review is an update of previous reviews [19–21], whereas for sleep questionnaires we included all relevant studies published up to now. Therefore, separate searches were carried out, i.e., one for physical activity and sedentary behavior, and one for sleep. The full search strategies can be found in Additional file 1.

For physical activity and sedentary behavior questionnaires, we searched the literature from December 2015 (i.e., last search period for sedentary behavior questionnaires [20]) until December 2019. In PubMed more overlap in time was maintained (search from May 2015), as our previous searches showed that the PubMed time filter can be inaccurate due to incorrect labeling of publication dates. For sleep questionnaires there was no lower limit for publication date, and databases were searched up until January 2020. A combined update search (i.e., physical activity, sedentary behavior and sleep) was completed on 6 January 2021.

Both search strategies focused on terms related to young children (e.g., infant, toddler, preschooler), proxy-report measures (e.g., questionnaire, proxy-report) and measurement properties (e.g., reliability, reproducibility, validity). For the physical activity and sedentary behavior search, these terms were used in AND-combination with terms related to physical activity (e.g., motor activity, exercise) OR sedentary behavior (e.g., stationary behavior, screen-time). For the sleep search, these terms were used in AND-combination with terms related to sleep (e.g., bedtime, nap). In all databases, studies in animals and children with a variety of diseases or disorders (e.g., autism, attention deficit disorder) were excluded using NOT-combinations.

### Inclusion and exclusion criteria

Studies were included when: (1) the study evaluated at least one of the measurement properties of a proxy-report questionnaire assessing physical activity, sedentary behavior and/or sleep; (2) the proxy-report questionnaire under study reported at least data on the duration and/or frequency of physical activity, sedentary behavior and/or sleep; (3) the study included apparently healthy children, born term (> 37 weeks), aged < 5 years or a wider age range with the results for 0- to 5-year-old children described separately; (4) the study was published in English in a peer-reviewed journal; and (5) the full-text was available.

Studies were excluded when (1) measurement properties were evaluated in a specific subpopulation or clinical sample (e.g., children with sleep problems); (2) construct validity was only evaluated by examining the relationship between the questionnaire and a non-similar construct (e.g., between physical activity and body mass index (BMI)); (3) structural validity and/or internal consistency were evaluated for questionnaires that represent a formative measurement model as these analysis are not relevant when the items of a scale or subscale form or cause the construct and are therefore not supposed to be correlated (e.g., items assessing different physical activities that together form the construct duration of total physical activity) [26, 27]; (4) responsiveness was evaluated without using a comparison measure to assess a questionnaire’s ability to detect change (e.g., accelerometer).

### Selection procedures

Articles were imported in EndNote X9.1, and subsequently duplicate articles were removed. Titles and abstracts of potentially relevant articles were scanned by two independent researchers (JA and TA) using Rayyan. Next, full texts were obtained and independently screened for eligibility by the same two researchers. Additionally, reference lists of all full-text articles were screened for additional studies. Disagreements were resolved through discussion.

### Inclusion of studies from previous reviews

To draw definite conclusions regarding the best available questionnaires, we also included studies from the three aforementioned reviews evaluating questionnaires assessing physical activity and sedentary behavior [19–21]. As these reviews were not restricted to questionnaires aimed to assess young children’s behaviors, only the studies that evaluated proxy-report questionnaires for children aged < 5 years were included, in line with our inclusion criteria.

### Data extraction

For all eligible studies, two independent reviewers (JA and either JG or AV) extracted data using a structured form. Disagreements were resolved through discussion. The following data was extracted: evaluated measurement properties, study population (i.e., population included in study), target population (i.e., population for which the questionnaire was developed), measurement instrument (i.e., name, construct(s), format), respondent, recall period, comparison method (in case of validity), time interval (in case of test-retest reliability), statistical method used, and results of the examined measurement properties (i.e., reliability, validity, responsiveness).

### Methodological quality assessment

Risk of bias was assessed by rating the methodological quality of included studies, using the COnsensus-based Standards for the selection of health Measurement Instruments (COSMIN) checklist [28]. Risk of bias refers to whether results are trustworthy based on the methodological quality of the study. For each examined measurement property, the study design requirements were rated as either very good, adequate, doubtful, or inadequate quality [28]. The lowest score counts method was applied, e.g., if one item was rated inadequate, the final methodological quality was rated as inadequate. For the rating of construct validity studies, the measurement properties of the comparator instrument(s) had to be taken into account. Measurement properties of accelerometers were evaluated using the systematic review of Lettink et al. [29]. An overview of the methodological quality ratings that could be given to different comparator instruments can be found in Additional file 2.

Two independent reviewers (JA and either TA, MC or AL) rated the methodological quality of the included studies. In the case of disagreement, a third researcher was consulted (either TA or MC).

### Rating study results

The study results of each measurement property were rated against the criteria for good measurement properties proposed in the COSMIN guideline (i.e., sufficient (+), insufficient (−), inconsistent (±) or intermediate (?)) [30]. Below is indicated for each measurement property which outcomes were considered sufficient (+). Outcomes were considered insufficient (−) when these criteria were not met, and intermediate (?) when not all necessary information was reported. Inconsistent (±) outcomes were only applicable for validity and reliability studies reporting multiple outcomes per questionnaire, and were rated as described below. The COSMIN methodology for evaluating content validity was used to rate the results of content validity studies [31]. For none of the included (versions of) questionnaires multiple studies on a measurement property were available. For this reason, quantitatively pooling or qualitatively summarizing study results was not possible.

#### Content validity

Content validity is defined as “the degree to which the content of a measurement instrument is an adequate reflection of the construct to be measured” [32]. Content validity consists of three components: relevance (e.g., relevant for construct and target population of interest), comprehensiveness (e.g., all key concepts are included), and comprehensibility (e.g., items, response options, and recall period are understood by the population of interest as intended) [31]. Relevance, comprehensiveness, and comprehensibility were rated using the criteria for good content validity [31]. All three components had to be rated as sufficient, in order to rate the overall content validity as sufficient [31].

#### Internal structure: structural validity, internal consistency, and cross-cultural validity

Internal structure refers to how the different items in a questionnaire are related, which is important to know for deciding how items might be combined into a scale or subscale [32]. To examine internal structure, three different measurement properties should be evaluated: (1) structural validity, (2) internal consistency and (3) cross-cultural validity. Structural validity and/or internal consistency are only applicable to questionnaires that represent a reflective measurement model, i.e., in which the items of the questionnaire are a reflection of the construct to be measured [27]. In a reflective model, the items are supposed to be correlated and interchangeable [26]. As a reflective measurement model is only applicable to questionnaires assessing sleep quality (i.e., items are a reflection of the construct sleep quality), structural validity and/or internal consistency were only evaluated for questionnaires assessing sleep quality.

Structural validity is defined as “the degree to which the scores of a questionnaire are an adequate reflection of the dimensionality of the construct to be measured” [32], and is usually evaluated by factor analysis. In case of exploratory factor analysis, structural validity outcomes were considered sufficient when factor loadings were ≥ 0.30 [33]. In case of confirmatory factor analyses, structural validity outcomes were considered sufficient when the comparative fit index or Tucker–Lewis index was > 0.95, mean square error of approximation was < 0.06, or standardized root mean residual was < 0.08 [30, 34].

Internal consistency refers to “the degree of the interrelatedness among the items”, and is often evaluated by Cronbach’s alpha [28, 32]. Internal consistency outcomes were considered sufficient if Cronbach’s alpha values were ≥ 0.70 and at least low quality of evidence for sufficient structural validity was provided (as rated by COSMIN guidelines) [30].

Cross-cultural validity or measurement invariance is defined as “the degree to which performance of the items of a translated or culturally adapted questionnaire are an adequate reflection of the performance of the items of an original version of the questionnaire” [32]. Cross-cultural validity is evaluated by group factor analyses or differential item functioning (DIF). When no important differences were found between group factors or DIF for group factors, cross-cultural validity/measurement invariance was considered sufficient [30].

#### Reliability

Reliability is defined as “the degree to which the measurement is free from measurement error” [32]. Reliability outcomes were considered sufficient if the intraclass correlation coefficients (ICC) or Kappa (K) values were ≥ 0.70 [30]. When Pearson or Spearman correlations were used to assess reliability, correlation coefficients had to be ≥0.80, because these correlations do not take systematic errors into account [26]. Most studies reported multiple correlations per questionnaire for test–retest reliability, e.g., separate correlations for each question or item. For this reason, an overall questionnaire rating was applied, i.e., incorporating all correlations, in order to obtain a final test–retest reliability rating for each questionnaire. When ≥75% of correlations were acceptable, a sufficient (+) rating was received, when ≥50% and < 75% of correlations were acceptable an inconsistent (±) rating was received, and an insufficient (−) rating was received when < 50% of correlations were acceptable.

#### Measurement error

Measurement error is “the systematic and random error of a score that is not attributed to true changes in the construct to be measured” [32]. Measurement error outcomes were considered sufficient when the standard error of measurement (SEM), smallest detectable change (SDC) (i.e., defined as “the smallest change that can be detected by the instrument, beyond measurement error” [26]) or limits of agreement (LoA) were smaller than the minimal important change (MIC) (i.e., defined as “the smallest change in score in the construct to be measured that is perceived as important by clinicians or relevant others” [26]) [30]. As the MIC was not defined, we could not give a final rating of the measurement error. Instead, we interpreted the measurement error outcomes per study.

#### Criterion and construct validity

Criterion validity is defined as “the degree to which the scores of a measurement instrument are an adequate reflection of a gold standard”. Criterion validity was considered sufficient when correlations with the gold standard were ≥ 0.70 [32]. We considered doubly labeled water as a reasonable gold standard for questionnaires aiming to assess physical activity energy expenditure. In addition, we considered polysomnography as a gold standard for questionnaires assessing sleep. Other comparators (e.g., accelerometers, diaries) were considered to reflect construct validity. Construct validity is “the degree to which the scores of a measurement instrument are consistent with (a priori drafted) hypotheses” (e.g., with regard to relationships to scores of other instruments, or differences between relevant groups) [32]. Since a priori drafted hypotheses for construct validity were often lacking, we formulated criteria with regard to the relationships with other instruments (e.g., accelerometers). Table 1 provides an overview of the criteria for evaluating the results of construct validity studies. These criteria were in line with previous reviews [20, 21], and are based on the similarity of the construct that is measured [30]. Additionally, we formulated criteria for studies that evaluated construct validity by comparing subgroups (i.e., children with and without sleep problems). The criteria were subdivided by level of evidence, level 1 indicating strong evidence, level 2 indicating moderate evidence, and level 3 indicating weak evidence. These levels of evidence indicate the confidence in the comparison method to accurately assess the relevant construct. Most studies reported multiple correlations with a comparator instrument, therefore, the same overall rating as used for reliability was applied for each questionnaire (i.e., sufficient (+), inconsistent (±), or insufficient (−)).

**Table 1:** A priori drafted hypotheses for the evaluation of construct validity of questionnaires assessing^a^ constructs of physical activity, sedentary behavior and/or sleep, subdivided by level of evidence^b^, and criteria for acceptable correlations/relationships with comparator instruments or subgroups^cd^

***Construct assessed***	***Level 1***	***Level 2***	***Level 3***
**Physical activity**			
Physical activity, all constructs (i.e., including at least indoor and outdoor activities of all intensities)	Acc. cut-point/algorithm for TPA ≥ 0.60	Acc. cut-point/algorithm for MPA, VPA or MVPA ≥0.40	Questionnaire or diary, corresponding constructs ≥0.70
Physical activity, not all constructs or timeframes	Acc. cut-point/algorithm for TPA; corresponding timeframe ≥0.60	Acc. cut-point/algorithm for TPA; total daytime ≥0.40 Acc. cut-point/algorithm for MPA and VPA ≥ 0.50	Questionnaire or diary, corresponding constructs ≥0.70
Physical activity, single constructs (e.g., outdoor play)		Acc. cut-point/algorithm for TPA ≥ 0.40 Acc. cut-point/algorithm for MPA and VPA ≥ 0.50	Questionnaire or diary, corresponding constructs ≥0.70
Vigorous physical activity	Acc. cut-point/algorithm for VPA ≥ 0.60	Acc. cut-point/algorithm for TPA ≥ 0.40	Questionnaire or diary, corresponding constructs ≥0.70
Moderate physical activity	Acc. cut-point/algorithm for MPA ≥ 0.60	Acc. cut-point/algorithm for TPA ≥ 0.40	Questionnaire or diary, corresponding constructs ≥0.70
Light physical activity	Acc. cut-point/algorithm for LPA ≥ 0.60	Acc. cut-point/algorithm for TPA ≥ 0.40	Questionnaire or diary, corresponding constructs ≥0.70
Moderate-to-vigorous physical activity	Acc. cut-point/algorithm for MVPA ≥0.60	Acc. cut-point/algorithm for TPA ≥ 0.40	Questionnaire or diary, corresponding constructs ≥0.70
**Sedentary behavior**			
Sedentary behavior, all constructs (i.e., including at least screen time and non-screen leisure time activities)		Acc. cut-point/algorithm for SB ≥ 0.60	Questionnaire or diary, corresponding constructs ≥0.70
Stationary behavior		Acc. cut-point/algorithm for SB ≥ 0.50	Questionnaire or diary, corresponding constructs ≥0.70
Screen time		Diary, logs ≥0.60	Questionnaire corresponding constructs ≥0.70
Sedentary behavior, not all constructs or time frames		Acc. cut-point/algorithm for SB; corresponding timeframe ≥0.60 Acc. cut-point/algorithm for SB; non-corresponding timeframe ≥0.50	Questionnaire or diary, corresponding constructs ≥0.70
**Sleep behavior**			
Sleep behavior, all constructs (i.e., including at least total sleep duration, sleep latency and night awakenings)	Videosomnography ≥0.60	Acc. cut-point/algorithm for sleep ≥0.40	Questionnaire, log, or diary, corresponding constructs ≥0.70 Acc. or diary not significantly different in the measured sleep construct. Discriminative validity: children without sleep problems score significantly better than children with sleep problems.
Sleep behavior, not all constructs or time frames	Videosomnography ≥0.60	Acc. cut-point/algorithm for sleep ≥0.40	Questionnaire, log, or diary, corresponding constructs ≥0.70 Acc. or diary not significantly different in the measured sleep construct. Discriminative validity: children without sleep problems score significantly better than children with sleep problems.
Sleep duration	Videosomnography ≥0.60	Acc. cut-point/algorithm for sleep ≥0.50	Questionnaire, log, or diary, corresponding constructs ≥0.70 Acc. or diary not significantly different in the measured sleep duration. Discriminative validity: children without sleep problems score significantly better than children with sleep problems.

Abbreviations: *Acc* accelerometer, *LPA* light physical activity, *MPA* moderate physical activity, *MVPA* moderate-to-vigorous physical activity, *TPA* total physical activity, *VPA* vigorous physical activity

^a^ Since a priori drafted hypotheses for construct validity were often lacking in included studies, we formulated criteria with regard to the relationships with other instruments (e.g., accelerometers) or subgroups

^b^ Level of evidence: level 1 indicating strong evidence, level 2 indicating moderate evidence, and level 3 indicating weak evidence. These levels of evidence indicate the confidence in the comparison method to accurately assess the relevant construct

^b^ The criteria for acceptable correlations with comparator instruments are based on the similarity of the construct that is measured

^d^ Table adapted from previous reviews by Hidding et al. [20, 21]

#### Responsiveness

Responsiveness is “the ability of a measurement instrument to detect change over time in the construct to be measured” [32]. As none of the included studies evaluated responsiveness of the questionnaire under study, responsiveness is not reported.

### Quality of evidence grading

The quality of evidence was graded using the Grading of Recommendations Assessment, Development and Evaluation (GRADE) approach, as proposed in the COSMIN guideline [30], indicating the trustworthiness of the measurement property results of questionnaires. The GRADE approach consists of four levels (i.e., high, moderate, low, very low), which depend on the presence of four risk factors: (1) risk of bias (i.e., the methodological quality of the studies), (2) inconsistency (i.e., unexplained inconsistency of results across studies), (3) imprecision (i.e., total sample size of the available studies), and (4) indirectness (i.e., evidence from different populations than the population of interest in this review) [30]. The quality of evidence was subsequently downgraded with one, two or three levels for each factor to moderate, low, or very low when there was risk of bias, inconsistency in results, a low sample size, or indirect results [30]. Because the COSMIN methodology has been updated since publication of the previous reviews, we re-evaluated all included studies from previous reviews on methodological quality and regraded the quality of evidence [30]. The grading of the quality of evidence was done for each measurement property and for each questionnaire separately by one researcher (JA). The ratings of measurement property outcomes (i.e., sufficient, insufficient, inconsistent, intermediate) were only presented in the results section for the measurement properties of questionnaires that received a high or moderate quality of evidence grading, as these results are considered most trustworthy. Consequently, a questionnaire can only be considered as valid/reliable when the quality of evidence is at least moderate, and the reliability/validity results are sufficient.

## Results

**Fig. 1:**
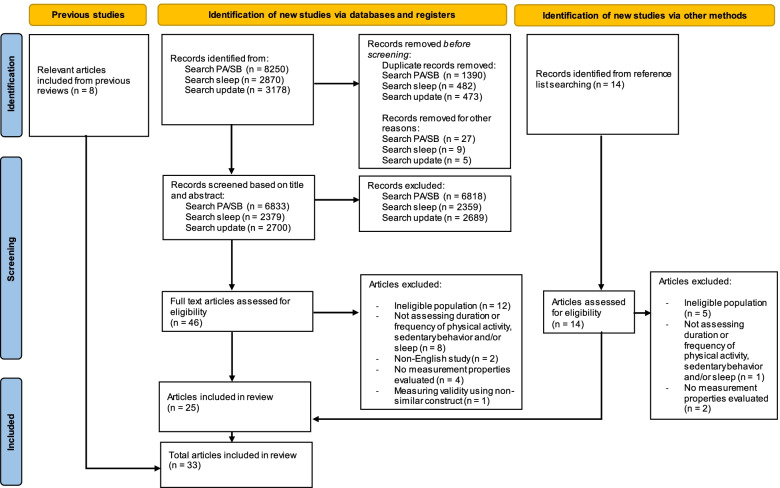
PRISMA flow diagram of study inclusion. A*bbreviations*: *PA* physical activity, *SB* sedentary behavior

Systematic literature searches using the PubMed, Embase, and SPORTDiscus databases yielded a total of 12,390 unique articles. After title and abstract screening, 46 full-texts were screened. Additionally, 14 articles were found through cross-reference searches. Therefore, a total of 60 full-text articles were assessed for eligibility, of which 25 were included. Additionally, we included 8 relevant articles from previous reviews [19–21], resulting in 33 articles that evaluated a total of 37 different (versions of) questionnaires (see Fig. 1 for the full selection process). Four questionnaires were (translated) versions of the Brief Infant Sleep Questionnaire (BISQ) [35–38], and three questionnaires were versions of the Children’s Sleep Habits Questionnaire (CSHQ) [39–41]. Table 2 presents the characteristics of all included questionnaires. Tables 3-6 summarize the results for content validity, internal consistency and structural validity, reliability, and criterion validity and construct validity.

**Table 2:** Characteristics of questionnaires assessing physical activity, sedentary behavior and/or sleep in 0- to 5-year-old children, sorted by behavior and target population

***Questionnaire Name***	***Behavior (PA, SB, ScT, S)*** ^b^	***Sub-constructs***	***Target population (age)*** ^c^	***Respondent***	***Recall period***	***Format: Dimensions (F, D, I)*** ^***d***^	***Format: Number of items***	***Format: Scores***
Sleep diary (nn) [38]	S	Night awakenings, wake after sleep onset (WASO), sleep latency, sleep onset, sleep duration	Infants (nr)	Parent	Current day	F, D	4 items	Continuous: time, duration (h/min), frequency (times per night)
Sleep diary by phone (nn) [42]	S	Sleep onset time, rise time, sleep period, number of night awakenings	Infants (nr)	Mother	Day	F, D	Unclear	Questions answered by phone
Electronic-diary (nn) [43]	S	Sleep during day (08-20 h), sleep during night (20-08 h), sleep over 24 h	Infants (nr)	Parent	Current day	D	Unclear	Unclear
Paper-diary (nn) [43]	S	Sleep during day (08-20 h), sleep during night (20-08 h), sleep over 24 h	Infants (nr)	Parent	Current day (5 min epochs)	D	Logline	Continuous: duration (h/min), derived from sleep state every 5 min
Sleep diary (nn) [44]	S	Night sleep duration, longest nighttime sleep, daytime sleep duration, night awakenings, sleep onset time, and sleep offset time	Infants (nr)	Parent	Current day (5 min epochs)	F, D	Logline	Continuous: time, duration (h/min), frequency (times per night), derived from sleep state every 5 min
Sleep survey (including items from the BISQ and CSHQ) [44]	S	Night sleep duration, longest nighttime sleep, daytime sleep duration, night awakenings, sleep onset time, and sleep offset time	Infants (nr)	Parent	Typical day	F, D	6 items	Continuous: time, duration (h/min), frequency (times per night)
Sleep and Settle Questionnaire (SSQ) [45]	S	Sleep patterns (number and duration of sleeps, night awakenings), time to settle to sleep, duration of crying Additionally: Temperament awake, confidence parent to get baby to sleep, attribution of unsettled infant behavior, whether parents are bothered by this	Infants (nr)	Primary caregivers	Last week	F, D	12 items Total questionnaire: 34 items	Continuous: duration (h/min), frequency (sleeps per day/times per night) Ordinal: 5-point Likert scale ranging from 1 ‘Didn’t bother me at all’ to 5 ‘Bothered me extremely’
Children’s Sleep Habits Questionnaire - Infant version (CSHQ-I) [39]	S	Bedtime resistance, sleep anxiety, daytime sleepiness, and positive sleep habits^e^	Infants (0–1 year old)	Mother	Typical week	F, D, I	31 items across 4 subscales: Bedtime resistance (11 items), Sleep anxiety (10 items), Positive sleep habits (6 items), and Daytime sleepiness (5 items)^e^	Continuous: time, duration (h/min) Ordinal: 3-point scale: “usually” (5–7 times per week); “sometimes” (2–4 times per week;) and “rarely” (0–1 time per week) (+not applicable)
Brief Infant Sleep Questionnaire (BISQ) [38]	S	Night awakenings, wake after sleep onset (WASO), sleep latency, sleep onset	Infants (nr)	Parent	Typical day	F, D	Unclear (other studies: 13 items)	Continuous: time, duration (h/min), frequency (times per night)
Expanded version of the Brief Infant Sleep Questionnaire (BISQ), Turkish version [37]	S	Nocturnal sleep duration (7 pm-7 am), daytime sleep durations (7 am-7 pm), number of night awakenings, duration of wakefulness during night, settling time Additionally:Method falling asleep, location of sleep, preferred body position, perception of sleep problems	Infants (nr)	Parent	Typical day	F, D	13 items (including 4 demographic)	Continuous: time, duration (h/min), frequency (times per night)
BISQ Spanisch version (BISQ-E) [35]	S	Time of sleep onset, duration of night-time sleep, duration of daytime sleep, total sleep duration, night-time awakenings, duration of night-time awakenings Additionally: Method falling asleep, location of sleep, preferred body position, perception of sleep problems	Infants and toddlers (3–30 months old)	Parent	Typical day	F, D	14 items (4 demographic, 10 sleep habits)	Continuous: time, duration (h/min), frequency (times per night)
BISQ (Nepali version) [36]	S	Nocturnal and daytime sleep duration, night awakenings, duration night wakefulness, sleep onset time, sleep settling time Additionally: Method falling asleep, location of sleep, preferred body position, perception of sleep problems	Infants and toddlers (0–3 years old)	Parent	Typical day	F, D	Unclear (other studies: 13 items)	Continuous: time, duration (h/min), frequency (times per night)
Sleep diary (nn) [46]	S	Sleep onset time, sleep offset time, nocturnal sleep time duration, number of night awakenings (> 5 min), wake minutes during nocturnal sleeping	Infants and toddlers (nr)	Mother	Current day (30 min epochs)	F, D	Unclear	Nominal: general questions per day Continuous: duration (h/min), derived from sleep state every 30 min
Sleep diary (nn) [47]	S	Sleep duration, sleep efficiency	Toddlers (2 years old)	Mother	Current day (30 min epochs)	D	Unclear	Continuous: duration (h/min), derived from sleep state every 30 min
Own questionnaire (nn) [48]	S	Get up time, bedtime, sleeping hours	Preschoolers (3–4 years old)	Parent	Current day	D	3 items	Continuous: time, duration (h/min)
Parent log (nn) [49]	S	Afternoon naps, bedtime, sleep latency, awakenings, sleep end time morning. Sleep onset time was calculated by adding sleep latency to the bedtime	Preschoolers (3–5 years old)	Parent (with input day care workers)	Current day	F, D	10 items (4 just before going to bed, and 6 first thing in the morning)	Continuous: time, duration (min) Ordinal: frequency of awakenings giving 4 options: 0, 1, 2, 3 or more
Children’s Sleep Habits Questionnaire (CSHQ) [41]	S	Quantitative: bedtime, wake-up time, daily sleep time, number and duration of night- awakenings Qualitative: Bedtime resistance, sleep onset delay, sleep duration, sleep anxiety, night-awakenings, parasomnias, obstructive sleep apnea, and daytime sleepiness^e^	Preschoolers (3–6 years old)	Parent	Typical week, typical weekday and weekend day	F, D, I	33 items across 8 subscales^e^	Continuous: time, duration (h/min), frequency (times per night) Ordinal: 3-point scale: “usually” (5–7 times per week); “sometimes” (2–4 times per week;) and “rarely” (0–1 time per week) (+not applicable)
Children’s Sleep Habits Questionnaire - Chinese version (CSHQ) [40]	S	Bedtime resistance, sleep onset delay, sleep duration, sleep anxiety, night-time awakenings, parasomnias, sleep disordered breathing, daytime sleepiness^e^	Preschoolers (3–6 years old)	Parent/Caregiver	Typical week	F, D, I	33 items across 8 subscales: Bedtime resistance (6 items), Sleep-onset delay (1 item), Sleep duration (3 items), Sleep anxiety (4 items), Night-time awakenings (3 items), Parasomnias (7 items), Sleep disordered breathing (3 items), and Daytime sleepiness (8 items)^e^	Continuous: time, duration (h/min) Ordinal: 3-point scale: “usually” (5–7 times per week); “sometimes” (2–4 times per week;) and “rarely” (0–1 time per week) (+not applicable)
Children’s ChronoType Questionnaire (CCTQ) - Japanese version [50]	S	Sleep-wake parameters, chronotype and morningness/eveningness (M/E)	Preschoolers (3–6 years old)	Parent	Typical weekday and weekend day	F, D	27 items across 3 subscales: Sleep–wake parameters (16 items), M/E (10 items), and Chronotype (1 item)	Continuous: time, duration (min), frequency (days per week) Nominal: yes/no Ordinal: different 5-point scales (e.g., ranging from Very difficult to Not at all a problem)
Children’s Sleep-Wake Scale (CSWS) [51]	S	Going to bed, falling asleep, arousing and awakening during the night, returning to sleep after waking during the night, waking in the morning	Children (2–8 years old)	Parent	Typical day	F, D, I	25 items across 5 subscales: Going to bed (5 items), falling asleep (5 items), arousing and awakening during the night (5 items), returning to sleep after waking during the night (5 items), waking in the morning (5 items)	Ordinal: 6-point scale (Never, Once in a while, Sometimes, Quite Often, Frequently - if not Always, and Always)
Technology Use Questionnaire (TechU-Q) [52]	ScT	Frequency, duration and purpose of use of multiple technology devices (television, computers, tablet computers, mobile phones, and electronic games)	Infants, toddlers, preschoolers (0–5 years old)	Parent/Caregiver	Typical weekday and weekend day	F, D	Unclear	Continuous: duration (h/min), frequency (times per week)
PREPS questionnaire [53]	ScT	Screen time Additionally: Children’s cognitions/personal attributes, parental cognitions, home and neighborhood environment	Toddlers (1–3 years old)	Parent	Typical weekday and weekend day	D	4 items Total questionnaire:83 items	Continuous: duration (h/min)
Outdoor time checklist [54]^a^	PA	Time playing outdoors	Toddlers and preschoolers (2–5 years old)	Parent	24 h (wake-up to bedtime)	D	2 items	Ordinal: 5-point scale with the following responses: 0 min; 1–15 min; 16–30 min; 31–60 min; and over 60 min
Parental report – outdoor time recall questionnaire [54]^a^	PA	Time playing outdoors	Toddlers and preschoolers (2–5 years old)	Parent	Typical week/weekend day in the last month	D	2 items	Continuous: time spent (min)
Modified Burdette proxy report [55]^a^	PA	Indoor and outdoor active play at family child care homes	Toddlers and preschoolers (2–5 years old)	Family Child Care Home Providers	Arrival until lunch time and lunch time until departure	D	1 item (2 times)	Ordinal: 5-point scale with the following responses: 0 min; 1–15 min; 16–30 min; 31–60 min; and over 60 min
Modified Harro proxy report [55]^a^	PA	Sitting, low-to moderate intensity indoor activities, low-to-moderate intensity outdoor activities, moderate-to-vigorous intensity indoor activities, and moderate-to-vigorous outdoor activities	Toddlers and preschoolers (2–5 years old)	Family Child Care Home Providers	Arrival until lunch and lunch until departure	D	5 items (2 times)	Continuous: t*ime spent* (min)
KidActive-Q [56]^a^	PA, ScT	Method of transportation to and from daycare, time spent outdoors, time spent watching television and/or playing computer games, and athletic activities performed on a regular basis	Toddlers and preschoolers (2–6 years old)	Parent	Usual, past few months	F, D	10–12 items	Nominal: yes/no Ordinal: different options for frequency and/or duration
Early Years Physical Activity Questionnaire (EY-PAQ) [57]	PA, SB	Various indoor and outdoor activities, which were grouped according to PA intensity (moderate-to-vigorous physical activity or sedentary time)	Toddlers and preschoolers (2–5 years old)	Parent	Typical week in previous month	F, D	16 items (MVPA: 10, ST: 6)	Ordinal: number of days per week, and duration giving 4 options: 1) up to 15 min/day; 2) 16–30 min/day; 3) 31–60 min/day; or 4) free-text for > 60 min/day
Preschool-aged Children’s Physical Activity Questionnaire (Pre-PAQ) [58]^a^	PA, SB	Time spent in car, time spent walking, activity, eating in front of television, attend organized physical activity, use of facilities to play or be physically active	Preschoolers (3–5 years old)	Parent/Guardian	Last week, yesterday, last weekend	F, D	Total questionnaire = 37 questions divided into 4 sections Number of items differs depending on answers	Continuous: duration (h/min) Nominal: yes/no Ordinal: 6-poimt scale ranging from daily to rarely
Children’s Physical Activity Questionnaire (CPAQ) [59]^a^	PA, SB	Various physical activity and sedentary activities, which were grouped in: sports, leisure time activities, and activities at school	Preschoolers (4–5 year old)	Parent	Last week	F, D	PA: 34 items SB: 15 items	Continuous: duration (h/min), frequency (times Mon-Fri/times Sat-Sun)
Healthy Kids [60, 61]	PA, ScT, S	TV and computer behavior, playing outside, and sleep Additionally: Diet and parenting practices	Toddlers and preschoolers of low income families (2–5 years old)	Parent/Caregiver	Typical week and day	F, D	Development study: PA: 4 items, ScT: 5 items, S: 1 item Total questionnaire: 45 items Selected in following study: PA: 1 item, ScT: 2 items, S: 1 item Total questionnaire: 19 items	Continuous: time, duration (h) Ordinal: 4-point scale ranging from no to every day
Physical activity questionnaire for parents of preschoolers in Mexico [62]^a^	PA, SB, S	Sleep and various indoor and outdoor activities, which were grouped according to PA intensity (sedentary, moderate, and vigorous)	Preschoolers (3–5 years old)	Parent	Typical week and day	F, D	PA: 3 items SB: 1 item S: 2 items Total questionnaire: 6 items	Continuous: duration (h/min), frequency (times per day/days per week) Ordinal: 5 categories ranging from 15 min. to more than 1 h
Energy balance-related behaviors (ERBs) selfadministered primary caregivers questionnaire (PCQ), from the ToyBox study [63]^a^	PA, SB, S	PA: duration sport, transport, playing outside; SB: watching TV/DVD/video, playing games on a computer or game console, quiet play; Sleep: night sleep duration, duration naps. Additionally: Sociodemographic and perinatal factors, water and beverages consumption, snacking	Preschoolers (nr)	Parent	PA: Yesterday/last weekday, last weekend day, usually. SB/S: Typical weekday and weekend day	D	PA: 27 items SB: 38 items S: 4 items Total questionnaire: 229 items	Continuous: duration (h/min) Ordinal: 10 categories ranging from Never to I don’t know (30 min and 1 h intervals)
Physical activity and sedentary behavior questionnaire (based on Canadian Health Measures Survey) [64]^a^	PA, SB, ScT	PA: time outside, time in a gymnasium, recess, unstructured free play; organized physical activities; SB: TV viewing, videos/DVD viewing, computer playing, game console playing, playing handheld devices, stroller time, traveling in motor vehicle	Infants, toddlers and preschoolers (0–6 years old)	Parent	Typical weekday and weekend day	D	PA: 5 items SB: 12 items	Continuous: time spent (min)
Surveillance of digital Media hAbits in earLy chiLdhood Questionnaire (SMALLQ™) [65]	PA, SB, ScT, S	Digital media use outside of pre-school/kindergarten; Non-digital behavior outside of pre-school/kindergarten: indoor and outdoor play, non-screen reading, and drawing; Day time naps, night-time sleep. Additionally: Digital media environment at home, parent digital media habits, perception, concern, awareness and practice of guidelines, demographic information	Toddlers and preschoolers (2–6 years old)	Parent	Typical weekday and weekend day	Digital media use: F,D; Non-digital behavior: D, Sleep: D, I	PA/SB/S: 4 items ScT: 4 items Total questionnaire: 25 items across 3 subscales: Digital media use (9 items), Non-digital behavior (3 items), Child and Parent information (13 items)	Continuous: duration (h/min), and percentage of time spent Ordinal: 6 point-scale ranging from never to 9 or more times a week
Family Health Survey (consists a.o. of Outdoor Playtime Recall, InFANT, POI.nz) [66]	PA, SB, ScT, S	Outdoor play, duration watching TV/DVD, using computer, using smartphone/tablet, playing videogame, sleep duration Additionally: Child parent interactions in relation to physical activity, screen time, and sleep	Preschoolers (3–6 years old)	Parent	Typical weekday and weekend day	D	PA: 1 item ScT: 4 items S: 2 items Total questionnaire: number of items differs depending on answers (max. 55 questions)	Continuous: time, duration (h/min)
Healthy Active Preschool Years (HAPPY) parent proxy report survey [67]	PA, SB, ScT, S	Participation in active transport, non-organized activities, participation in organized activities, attendance at playgroup, usual participation in screen behaviors, quiet play & imaginary games, time spent playing outdoors, night & day sleep time Additionally: Demographic and family profile; child personality, preferences and constraints; parental influence; rules and boundaries, social interaction and support; modeling of physical activity; home physical environment; neighborhood physical environment	Preschoolers (3–5 years old)	Parent	Typical week, typical weekday and weekend day	F, D	PA/SB/ScT: 28 items S: 2 items Total questionnaire: 230 items	Continuous: duration (h) and frequency (times per week) Ordinal: never-daily Nominal: yes/no

*Abbreviations: BISQ* Brief Infant Sleep Questionnaire, *D* duration, *F* frequency, *h* hour, *I* intensity, *nn* no name, *nr* not reported, M/E morningness/eveningness, *MVPA* moderate-to-vigorous activity, *PA* physical activity, *PREPS* Parents’ Role in Establishing healthy Physical activity and Sedentary behaviour habits, *S* sleep, *SB* sedentary behavior, *ScT* screen time, *ST* sedentary time, *TV* television

^a^ Questionnaire included from previous review

^b^ Behaviors: physical activity (PA), sedentary behavior (SB), screen time (ScT) and/or sleep (S)

^c^ Infants: 0**–**1 year old, Toddlers: 1**–**3 years old, Preschoolers: 3**–**5 years old

^d^ Dimensions: frequency (F), duration (D), and/or intensity (I) of physical activity, sedentary behavior and/or sleep. Quality of sleep is also considered as an intensity

^e^ Note that some of the items included in this questionnaire might reflect indicators of sleep disorders, rather than sleep quality or duration/frequency of sleep

**Table 3:** Content validity of physical activity, sedentary behavior and/or sleep questionnaires, including methodological quality, result rating and quality of evidence

***Questionnaire***	***Study population***	***Methodological quality*** ^***a***^	***Results (rating)*** ^b^			***Overall rating*** ^c^ ***& evidence grading*** ^d^
			*Relevance*	*Comprehensiveness*	*Comprehensibility*	
Children’s Sleep-Wake Scale (CSWS) [51]	n = 9 pediatric sleep experts *n* = 30 primary caregivers of 2- to 5-year-old children	Doubtful	Pediatric sleep experts quantitatively evaluated the content relevance of each item. Items of low content validity were removed which resulted in a CVI of 0.93 for the entire instrument. (+)	Pediatric sleep experts quantitatively evaluated the comprehensiveness of the entire scale as a measure of children’s behavioral sleep quality. Items of low content validity were removed which resulted in a CVI of 0.93 for the entire instrument. (+)	Primary caregivers provided qualitative feedback on the clarity of directions and items, suitability of the scaling method, and approximate time to complete administration. Following scale revisions, pediatric sleep experts quantitatively evaluated the clarity and conciseness of the administration directions and items. Items of low content validity were removed which resulted in a CVI of 0.93 for the entire instrument. (?)	?^d^Low
BISQ (Nepali version) [36]	*n* = 15 parents with child aged < 3 years old	Doubtful	na	na	The respondents had no difficulties in understanding the questionnaire, their answers were appropriate and none of the parents returned the questionnaire for any clarification. (?)	?Low
Surveillance of digital Media hAbits in earLy chiLdhood Questionnaire (SMALLQ™) [65]	n = 4 experts *n* = 137 teachers, parents, and school leaders of preschool centers and kindergartens in Singapore	Doubtful	Experts, including parents, independently reviewed early versions of the questionnaire and guided the development of new questions that were contextually relevant, of concern and interest, and useful. (+)	na	Cognitive load and the ease of understanding of the questionnaire items were tested using a focus group, and, where necessary, the questionnaire items were refined and reorganized. When pilot-testing, average time taken to complete the questionnaire was 20**–**30 min. Based on qualitative feedback, amendments were made to the questionnaire to enhance its utility and ease of response for participants of the survey. (+)	?Low
Healthy Kids [60]	*n* = 77 ethnically diverse low-income parents	Doubtful	Relevant items for this tool were identified from results of comprehensive literature reviews for the broad determinants of obesity, corresponding behaviors and survey items. (+)	na	Cognitive interviews provided contextual rich qualitative data for instrument development, including how respondents interpreted text and photographs and their recommendations for changes to improve understanding, consistency of interpretation, and appeal by limited literacy readers. Researchers agreed the message was consistent with the original intent for each item. (+)	?Low
Family Health Survey (consists a.o. of Outdoor Playtime Recall, InFANT, POI.nz) [66]	*n* = 24 parents of children in ECE (from urban and rural North-eastern Brazil)	Doubtful	na	na	In the cognitive interviews, parents understood most items, but requested modifications to the formatting of the questionnaire, recall period, and the wording of a small number of items. The process of translation and cognitive interviews conducted in Brazilian families resulted in an appropriate cultural adaptation of scales measuring children’s movement behaviors and parenting practices. (+)	? Low
Technology Use Questionnaire (TechU-Q) [52]	*n* = 94 parents *n* = 10 experts (in research of technology use by children, measurement and activity and task behaviors, and childcare professionals)	Doubtful	na	Experts were asked if any common technology devices were not included. Overall, experts agreed with the measured constructs and questions. (+)	Parents found the questions appropriate and no major changes were suggested based on parent feedback. Experts commented on the content validity and made suggestions to wording and question structure. Questions were modified based on substantial and consensus comments. (+)	?Low

*Abbreviations* **:** *BISQ* Brief Infant Sleep Questionnaire, *COSMIN* COnsensus-based Standards for the selection of health Measurement Instruments, *CVI* content validity index, *na* not assessed

^a^ Methodological quality based on the COSMIN risk of bias checklist

^b^ Result rating for relevance, comprehensiveness, comprehensibility based on the COSMIN methodology for content validity

^c^ Overall rating of content validity results based on the COSMIN methodology for content validity. If not all ratings of content validity (i.e., relevance, comprehensiveness, comprehensibility) were available, an intermediate (?) score was given

^d^ Quality of evidence grading based on the COSMIN methodology for content validity

**Table 4:** Internal consistency and structural validity of sleep questionnaires, including methodological quality, result rating and quality of evidence

***Questionnaire***	***Study population*** ^a^	***Internal consistency***			***Structural validity***		
		***Methodological quality*** ^***b***^	***Results***	***Rating & Evidence grading*** ^c^	***Methodological quality*** ^b^	***Results***	***Rating & Evidence grading*** ^***c***^
Children’s Sleep-Wake Scale (CSWS) [51]	*n* = 543 Age = 4.9 ± 2.0 years (range 2–8) Sex = unknown	Very good	Cronbach’s α for the total scale was 0 .89. Cronbach’s α for the subscales were: Going to Bed (α = 0.88), Falling Asleep (α = 0.83), Maintaining Sleep (α = 0.81), Reinitiating Sleep (α = 0.81), and Returning to Wakefulness (α = 0.91)	+ High			
Children’s Sleep Habits Questionnaire - Chinese version (CSHQ) [40]	*n* = 2816 Age = 4.82 ± 1.06 years Sex = 46.9% girls	Very good	Cronbach’s α for the total scale was 0.72. Cronbach’s α for the subscales were: Sleep duration (α = 0.46), Sleep disordered breathing (α = 0.54), Sleep anxiety (α = 0.57); Parasomnias (α = 0.58, and Daytime sleepiness (α = 0.63)	– High	Adequate	EFA: model was adjusted to acceptable eight-factor structure; CFA: comparative fit index = 0.91, Tucker–Lewis index = 0.90, and standardized root mean residual = 0.03	+Moderate
Children’s Sleep Habits Questionnaire - Infant version (CSHQ-I) [39]	*n* = 299 Age = 2–12 months Sex = 46.8% girls	Very good	Cronbach’s α for the total scale was 0.78. Cronbach’s α for the subscales were: Bed-time Resistance (α = 0.77), Sleep Anxiety (α = 0.66), Positive Sleep Habits (α = 0.58), and Daytime Sleepiness (α = 0.52)	– High	Adequate	EFA: after removing items with factor loadings < 0.30, 33 items remained	+Moderate

*Abbreviations*: *COSMIN* COnsensus-based Standards for the selection of health Measurement, CFA confirmatory factor analysis, EFA exploratory factor analysis

^a^ Age presented as mean age ± SD (range)

^b^ Methodological quality based on the COSMIN risk of bias checklist

^c^ Result rating and quality of evidence grading based on the COSMIN guidelines

**Table 5:** Reliability of physical activity, sedentary behavior and/or sleep questionnaires, sorted by quality of evidence, result rating and methodological quality

***Questionnaire***	***Reliability***				
	***Study population*** ^***b***^	***Time interval***	***Methodological quality*** ^c^	***Results***	***Rating*** ^d^ ***& evidence grading*** ^***e***^
Preschool-aged Children’s Physical Activity Questionnaire (Pre-PAQ) [58]^a^	*n* = 103 Age = 3.80 ± 0.74 years Sex = 48% girls	1**–**2 weeks	Adequate	Pre-PAQ level 1–2: ICC = 0.44; Pre-PAQ level 3: ICC = 0.53; Pre-PAQ level 4: ICC = 0.44; Pre-PAQ level 5: ICC = 0.64; Time child spent in car: ICC = 0.31–0.63; Child’s activity nature: ICC = 0.87–0.93; Involvement in organized activities: K = 0.95, Time spent in organized activities: ICC = 0.96–0.99; Use of neighborhood facilities for activity: K = 0.70–0.80	– Moderate
Early Years Physical Activity Questionnaire (EY-PAQ) [57]	*n* = 109 Age = 3.3 ± 0.8 years Sex = 53% girls	7.2 days (5**–**12 days)	Adequate	MVPA: ICC = 0.35 (95% CI 0.17–0.50); ST: ICC = 0.47 (95% CI 0.30–0.61)	–Moderate
PREPS questionnaire [53]	*n* = 118 Age = 19.3 ± 2.7 months Sex = 48% female	7 days	Doubtful	Screen time: ICC = 0.82	+Low
Energy balance-related behaviors (ERBs) selfadministered primary caregivers questionnaire (PCQ), from the ToyBox study [63]^a^	*n* = 93 preschooler Age = unknown Sex = unknown	2 weeks	Adequate	ICC (95% CI) = Sports: time per week = 0.93 (0.85–0.97), type of sport = 0.71 (0.46–0.86); Active/passive transport: travel forth = 0.91 (0.87–0.94), time = 0.82 (0.73–0.88), travel home = 0.88 (0.82–0.92), time = 0.89 (0.83–0.93); Computer use: weekdays = 0.72 (0.60–0.80), weekend days = 0.81 (0.73–0.87); TV viewing: weekdays = 0.67 (0.54–0.77), weekend days = 0.67 (0.53–0.77); Quiet play: weekdays = 0.42 (0.24–0.58), weekend days = 0.50 (0.33–0.64); Night sleep duration: weekdays = 0.68 (0.55–0.78), weekend days = 0.69 (0.56–0.78); Naps: weekdays = 0.62 (0.48–0.73), weekend days = 0.70 (0.58–0.79)	±Low
Children’s Sleep Habits Questionnaire - Chinese version (CSHQ) [40]	*n* = 82 Age = unknown Sex = unknown	2 weeks	Adequate	ICC = Full-scale = 0.77, Sleep duration = 0.38, Bedtime resistance = 0.78, Night-time awakenings = 0.54, Parasomnias = 0.56, Sleep onset delay = 0.57, Daytime sleepiness = 0.60, Sleep disordered breathing = 0.67, Sleep anxiety = 0.76	–Low
Children’s Sleep Habits Questionnaire - Infant version (CSHQ-I) [39]	*n* = 98 (completing all 3 times points) Age = 2**–**12 months Sex = unknown	3 months (2 times)	Doubtful	CSHQ-I total scale scores: between 0 and 3 months and 3–6 months (r = 0.64), between 3 and 6 months and 6–12 months (r = 0.73); The subscales Bedtime Resistance, Sleep Anxiety, and Positive Sleep Habits: between 0 and 3 months and 3–6 months (r range = 0.48–0.56), between 3 and 6 months and 6–12 months (r range = 0.43–0.73); The subscale Daytime Sleepiness: between 0 and 3 months and 3–6 months (r = 0.08), between 3 and 6 months and 6–12 months (r = 0.23)	–Low
Healthy Kids [61]	Year 1: *n* = 133; Year 2: *n* = 98 Sex = unknown	12 weeks	Doubtful	Television: r = 0.44, CV = 24%; Computer games: r = 0.34, CV = 15%; Play: r = 0.32, CV = 31%; Bedtime: CV = 38%	–Low
KidActive-Q [56]^a^	*n* = 20 Age = 4.2 ± 1.3 years (range: 2–6) Sex = 50% girls	3 weeks	Adequate	ICC (95% CI): Watching TV = 0.85 (0.72–0.97); Overall PA level = 0.66 (0.41–0.91); Time spent outdoors = 0.60 (0.31–0.88)	+ Very low
BISQ Spanisch version (BISQ-E) [35]	*n* = 60 Age = 3–30 months Sex = unknown	10 days (8–22 days)	Doubtful	Inter-observer reliability: K = 0.94 (95% CI 0.85–1.00); Test - retest reliability: bed-time (r = 0.74), hours of night-time sleep (r = 0.88), hours of daytime sleep (r = 0.90) and number of awakenings (r = 0.88)	+ Very low
Physical activity questionnaire for parents of preschoolers in Mexico [62]^a^	*n* = 21 Age = 3–5 years old Sex = unknown	1 week	Doubtful	Duration low activity: r = 0.86; Duration moderate activity: r = 0.79; Duration vigorous activity: r = 0.94; Overall activity: r = 0.97	+ Very low
Technology Use Questionnaire (TechU-Q) [52]	n = 22–27 Age = unknown Sex = unknown	2 weeks	Adequate	ICC for weekday, weekend day and total: TV = 0.58, 0.64, 0.72; Desktop = 0.91, 0.96, 0.92; Laptop = 0.20, 0.35, 0.25; Tablet = 0.50, 0.93, 0.75; Mobile = 0.92, 0.88, 0.90; Total tech = 0.70, 0.70, 0.71	±Very low
Healthy Active Preschool Years (HAPPY) parent proxy report survey [67]	*n* = 43 Age = unknown Sex = unknown	24 days (14–50 days)	Adequate	ICC for summed items/continuous variables: Child’s sleep: individual variables = 0.75–0.78, combined sum = 0.72; Active transport = 0.75; Non-organized activities = 0.63: Total organized activities = 0.70; Usual participation in screen behaviors: Individual items = 0.31–0.84 (week days); 0.05–0.75 (weekend days). Summed items: weekly TV = 0.69, weekly e-games = 0.55, weekly computer = 0.51, weekly Wii/Eye Toy = 0.23, total weekly SBE time = 0.68, total week day SBE time = 0.44, total weekend SBE time = 0.70; Quiet play/imaginary games: 0.30–0.50 (weekday), 0.34–0.40 (weekend day), 0.37–0.63 (total weekly); Summed items: weekly quiet play = 0.63, weekly imaginary games = 0.37 K (% agreement) for categorical variables: Child’s PA & SB = − 0.02 to 0.90 (50.07–95.84); Organized activities: participation = 0.66–0.84 (91.49–100.0), frequency = 0.38–0.70 (88.9–93.62); Playgroup attendance: participation = 0.91 (95.74); frequency = 0.77 (93.33); Screen behaviors: participation = 0.52–1.00 (82.22–100.0)	±Very low
Children’s Physical Activity Questionnaire (CPAQ) [59]^a^	*n* = 27 Age: 4.9 ± 0.7 years (range: 4–5 years) Sex: 38% girls	7 days	Adequate	MVPA: ICC = 0.39; PAEE: ICC = 0.25	–Very low
Expanded version of the Brief Infant Sleep Questionnaire (BISQ), Turkish version [37]	*n* = 30 Age = 9 months Sex = unknown	3 weeks	Doubtful	Nocturnal sleep duration (r = 0.71), daytime sleep duration (r = 0.73), number of night awakenings (r = 0.82), duration of nocturnal wakefulness (r = 0.77), nocturnal sleep onset time (r = 0.81), and settling time (r = 0.63)	–Very low
Children’s Sleep-Wake Scale (CSWS) [51]	*n* = 36 Age = 4.4 ± 2.1 years Sex = unknown	1 month	Doubtful	CSWS total (r = 0.85), going to bed (r = 0.84), falling asleep (r = 0.78), maintaining Sleep (r = 0.75), reinitiating Sleep (r = 0.67), and returning to wakefulness (r = 0.70)	–Very low
Sleep and Settle Questionnaire (SSQ) [45]	*n* = 20 Age = unknown Sex = unknown	7–14 days	Doubtful	Morning sleeps (r = 0.38), afternoon sleeps (r = 0.34), night sleeps (r = 0.14), number of daytime naps (r = 0.40), number awakenings at night (r = 0.43), how long to settle in the day (r = 0.01), how long to settle in evening (r = 0.26), how long to settle at night (r = 0.52)	–Very low

*Abbreviations: BISQ* Brief Infant Sleep Questionnaire, *CI* confidence interval, *COSMIN* COnsensus-based Standards for the selection of health Measurement Instruments, *ICC* intraclass correlation name, *K* kappa, *M* mean, *MVPA* moderate-to-vigorous physical activity, *PA* physical activity, *PREPS* Parents’ Role in Establishing healthy Physical activity and Sedentary behavior, *r* correlation coefficient (Pearson or Spearman), *SB* sedentary behavior, *SBE* screen behavior entertainment*, ST* sedentary time

^a^ Questionnaire included from previous review

^b^ Age presented as mean age ± SD (range)

^c^ Methodological quality based on the COSMIN risk of bias checklist

^d^ Result rating: + indicates ≥75% acceptable correlations; +/− indicates ≥50 to < 75% acceptable correlations; − indicates < 50% acceptable correlations

^e^ Quality of evidence grading based on the COSMIN guidelines

**Table 6:** Criterion/construct *v*alidity of physical activity, sedentary behavior and/or sleep questionnaires, sorted by quality of evidence, level of evidence, result rating and methodological quality

***Questionnaire***	***Study population*** ^***b***^	Comparison measure	***Methodological quality*** ^***c***^	***Results***	***Rating*** ^d^ ***, level of evidence*** ^***e***^ ***& evidence grading*** ^***f***^
Early Years Physical Activity Questionnaire (EY-PAQ) [57]	*n* = 196 Age = 3.2 ± 0.8 years Sex = 49.5% girls	Accelerometer (Actigraph, GT3X+, right hip, 5 s (Costa)/15 s (Pate) epoch, Costa (SB) / Pate (PA) cut-points [68, 69])	Doubtful (PA) / Adequate (SB)	MVPA: MD = 7.1 min/day (LoA = 85.9 ± 200.1); r = 0.03 (no boundaries); r = 0 .30 (boundaries applied) ST: MD = 87.5 min/day (LoA = 376.6 ± 192.7); r = 0.02 (no boundaries); r = 0.19 (boundaries applied)	PA: – (Level 1) Low SB: – (Level 2) Moderate
Sleep diary by phone (nn) [42]	*n* = 90 Age: = 3.61 months (range 2.67–5.17 months) Sex = 43% girls	- Videosomnography (four infrared, high-definition, color Hikvision cameras with internal microphones) - Accelerometer (Actiwatch-2, left ankle, 15 s epoch, default wake threshold value = .888 * average activity count)	Adequate (Vid) / Doubtful (Acc)	Videosomnography correlations: Sleep onset time (r = 0.84), rise time (r = 0.74), sleep period (r = 0.81), night awakenings (r = 0.37); Videosomnography MD (min): Sleep onset time = − 0:23:51, rise time = 0:13:19, sleep period = 31.99, night awakenings = − 1.38; Accelerometer correlations: Sleep onset time (r = 0.91), rise time (r = 0.84), sleep period (r = 0.90), night awakenings (r = 0.51) Accelerometer MD (min): Sleep onset time = 0:06:17, rise time = 0:15:08, sleep period = 8.41, night awakenings = −.26;	Vid: + (Level 1) Low Acc: + (Level 2) Very low
Preschool-aged Children’s Physical Activity Questionnaire (Pre-PAQ) [58]^a^	*n* = 67 Age = 3–5 years Sex = 38% girls	Accelerometer (Actigraph, uni-axial MTI 7164, right hip, 15 s epoch, Sirard/Reilly cut-points [70, 71])	Adequate^g^ (both PA and SB)	Level 1–2 Pre-PAQ vs Stationary: MD = 7.6, LoA = [− 141.3, 156.4], r = 0.25; Level 1–2 Pre-PAQ vs Sedentary (Reilly): MD = − 208.6, LoA = [− 349.8, − 67.5], r = 0.28; Level 1–2 Pre-PAQ vs Sedentary (Sirard): MD = − 235.4, LoA = [− 383.1, − 87.7], r = 0.19; Level 3 Pre-PAQ vs LPA (Sirard): MD = − 4.8, LoA = [− 105.4, 96.0], r = − 0.07; Level 4 Pre-PAQ vs MPA (Sirard): MD = 48.2, LoA = [− 24.9, 121.3], r = 0.13; Level 5 Pre PAQ vs VPA (Sirard): MD = 1.9, LoA = [− 37.5, 41.3], r = 0.17; Level 4–5 Pre-PAQ vs MVPA (Sirard): MD = 50.1, LoA = [− 42.9, 143.1], r = 0.17; Level 3–5 Pre-PAQ vs LMVPA (Sirard): MD = 45.2, LoA = [− 103.6, 194.1], r = 0.05; Level 3–5 Pre-PAQ vs Non-sedentary (Reilly): MD = 20.9, LoA = [− 121.9, 163.7], r = 0.16	PA: – (Level 1) Low SB: – (Level 2) Low
Modified Burdette proxy report [55]^a^	*n* = 107 Age = 3.4 ± 1.2 years Sex = unknown	Accelerometer (Actigraph, GTM1, right hip, 15 s epoch, Pate cut-points [72])	Doubtful	PA: vs. total PA min/day: r = 0.30; vs. MVPA min/day: r = 0.34	– (Level 1) Low
Modified Harro proxy report [55]^a^	*n* = 131 Age = 3.8 ± 1.3 years Sex = unknown	Accelerometer (Actigraph, GTM1, right hip, 15 s epoch, Pate cut-points [72])	Doubtful	MVPA: vs. MVPA min/day: r = 0.10; vs. total PA min/day: r = 0.09	– (Level 1) Low
Electronic-diary (nn) [43]	*n* = 90 Age = 5.0 ± 2.0 months (range 1–9) Sex = 50% girls	Accelerometer (MicroMini Motionlogger Actigraph, ankle, 60s epoch, Sadeh algorithm [73])	Adequate	Sleep percentage over 24 h (r = 0.41), sleep percentage during the day (r = 0.65), sleep percentage at night (r = 0.64)	+ (Level 2) Low
Paper-diary (nn) [43]	*n* = 95 Age = 5.0 ± 2.0 months (range 1–9) Sex = 47% girls	Accelerometer (MicroMini Motionlogger Actigraph, ankle, 60s epoch, Sadeh algorithm [73])	Adequate	Sleep percentage over 24 h (r = 0.57), Sleep percentage during the day (r = 0.47), Sleep percentage at night (r = 0.70)	+ (Level 2) Low
Sleep diary (nn) [44]	*n* = 314 Age = 6.4 ± 0.6 months Sex = 51% girls	- Accelerometer (Philips Actiwatch 2, left ankle, 30s epoch, wake threshold value = 80 [74]) - Questionnaire (Sleep survey)	Doubtful (both methods)	Accelerometer ICC: Total night sleep duration = 0.51, longest night sleep = 0.40, total day sleep duration = 0.53; Accelerometer MD (95% CI): Total night sleep duration = − 42.9 (− 50.6 – − 35.2), longest night sleep = 58.2 (43.4–73.0), total day sleep duration = − 30.8 (− 35.9 – − 25.6); Questionnaire ICC: Total night sleep duration = 0.44, longest night sleep = 0.55, total day sleep duration = 0.33, sleep onset time = 0.31, sleep offset time = 0.10; Questionnaire MD (95% CI): Total night sleep duratio*n* = 22.9 (14.0–31.7), longest night sleep = 1.8 (− 17.2–13.6), total day sleep duration = − 1.0 (− 10.3–8.3), sleep onset time = 0.2 (0.0, 0.5), sleep offset time = − 0.4 (− 0.6 – − 0.1)	Acc: + (Level 2) Low QA: – (Level 3) Low
Sleep diary (nn) [38]	Data were collected at 3 (*n* = 226, girls = 49.5%), 6 (*n* = 191), 12 (*n* = 172), and 18 months (*n* = 150) postpartum.	- Accelerometer (micromotion logger sleep watch, 60s epoch, Sadeh algorithm [75]) - Questionnaire (BISQ)	Doubtful (both methods)	Accelerometer correlations at 3, 6, 12 and 18 months: Night awakenings: 0.58, 0.56, 0.40, 0.27; WASO = 0.72, 0.69, 0.63, 0.61; Sleep duration = 0.87, 0.94, 0.61, 0.78; Sleep onset = 0.88, 0.89, 0.89, 0.84; BISQ correlations at 3, 6, 12 and 18 months: Night awakenings: 0.70, 0.76, 0.68, 0.59; WASO = 0.33, 0.34, 0.54, 0.69; Sleep latency = 0.40, 0.43, 0.59, 0.63; Sleep onset = 0.82, 0.82, 0.81, 0.78	Acc: + (Level 2) Low QA: – (Level 3) Low
Brief Infant Sleep Questionnaire (BISQ) [38]	Data were collected at 3 (n = 226, girls = 49.5%), 6 (*n* = 191), 12 (*n* = 172), and 18 months (n = 150) postpartum.	- Accelerometer (micromotion logger sleep watch, 60s epoch, Sadeh algorithm [75]) - Sleep diary	Doubtful (both methods)	Accelerometer correlations at 3, 6, 12 and 18 months: Night awakenings: 0.53, 0.48, 0.27, 0.06; WASO = 0.22, 0.15, 0.31, 0.39; Sleep onset = 0.74, 0.75, 0.78, 0.72 Diary correlations at 3, 6, 12 and 18 months: Night awakenings: 0.70, 0.76, 0.68, 0.59; WASO = 0.33, 0.34, 0.54, 0.69; Sleep latency = 0.40, 0.43, 0.59, 0.63; Sleep onset = 0.82, 0.82, 0.81, 0.78	Acc: ± (Level 2) Low Diary: – (Level 3) Low
Sleep survey (including items from the BISQ and CSHQ) [44]	n = 314 Age = 6.4 ± 0.6 months Sex = 51% girls	- Accelerometer (Philips Actiwatch 2, left ankle, 30s epoch, wake threshold value = 80 [74]) - Sleep diary	Doubtful (both methods)	Accelerometer ICC: Total night sleep duration = 0.34, longest night sleep = 0.17, total day sleep duration = 0.25, sleep onset time = 0.23, sleep offset time = 0.08; Accelerometer MD (95% CI): Total night sleep duration = − 67.1 (− 76.3 – − 57.9); longest night sleep = 58.2 (39.9–76.4), total day sleep duration = − 28.9 (− 38.1 – − 19.7), sleep onset time = − 0.3 (− 0.6 – − 0.1), sleep offset time = 0.4 (0.1–0.6); Sleep diary ICC: Total night sleep duration = 0.44, longest night sleep = 0.55, total day sleep duration = 0.33, sleep onset time = 0.31, sleep offset time = 0.10; Sleep diary MD (95% CI): Total night sleep duration = 22.9 (14.0–31.7), longest night sleep = 1.8 (− 17.2–13.6), total day sleep duration = − 1.0 (− 10.3–8.3), sleep onset time = 0.2 (0.0–0.5), sleep offset time = − 0.4 (− 0.6 – − 0.1)	Acc: – (Level 2) Low Diary: – (Level 3) Low
Parental report – outdoor time checklist [54]^a^	*n* = 250 Age: 44 months (29–52 months) Sex: 43% girls	- Accelerometer (RT3 tri-axial research tracker, waist worn, 60s epoch) - Questionnaire (Outdoor time recall questionnaire)	Doubtful (both methods)	Accelerometer: r = 0.33; Recall questionnaire: r = 0.57	Acc: – (Level 2) Low QA: – (Level 3) Low
Parental report – outdoor time recall questionnaire [54]^a^	n = 250 Age: 44 months (29–52 months) Sex: 43% girls	- Accelerometer (RT3 tri-axial research tracker, waist worn, 60s epoch) - Questionnaire (Outdoor time checklist)	Doubtful (both methods)	Accelerometer: r = 0.20; Checklist questionnaire: r = 0.57	Acc: – (Level 2) Low QA: – (Level 3) Low
Healthy Kids [61]	*n* = 176 Age = 3.3 ± 1.6 years Sex = 42% girls	Parent reported physical, screen, and sleep activity using 3 days of 36-h activity logs	Doubtful	Television: r = − 0.53; Computer games: r = − 0.50; Play: r = 0.21, Bedtime: r = 0.22	– (Level 3) Low
Children’s Sleep Habits Questionnaire - Infant version (CSHQ-I) [39]	*n* = 299 Age = 2–12 months Sex = 46.8% girls	Sleep diary (Infant Sleep Chronogram)	Doubtful	Sleep hours (r = − 0.36), awake hours (r = 0.26), night-time awakenings (r = 0.04), latency to sleep (r = 0.14), longest sleep period (r = − 0.36)	– (Level 3) Low
Children’s Physical Activity Questionnaire (CPAQ) [59]^a^	n = 27 Age: 4.9 ± 0.7 years (range 4–5) Sex: 38% girls	- Doubly labeled water (DLW) - Accelerometer (Actigraph, model 7165, hip left/right randomized, 60s epoch, Treuth/Freedson cut-points [76, 77])	Very good (DLW) / Doubtful^g^ (Acc)	DLW: PAEE: r = 0.22; Accelerometer (both cut-points): MVPA: r = 0.42	DLW: – (Level 1) Low Acc: – (Level 1) Very low
Physical activity questionnaire for parents of preschoolers in Mexico [62]^a^	*n* = 35 Age = 4.4 ± 0.7 years Sex = 51% girls	Accelerometer (Actigraph, GT1M, right hip, 15 s epoch, Sirard/Pate cut-points [70, 72])	Adequate^g^ (both PA and SB)	Sirard cut-points: SB vs. % of time in SB: 0.35; MPA vs. % of time in MPA: r = − 0.23; VPA vs. % of time in VPA: r = 0.53; MVPA vs. % of time in MVPA: 0.49 Pate cut-points: SB vs. % of time in SB: 0.34; MPA vs. % of time in MPA: r = − 0.07; VPA vs. % of time in VPA: r = 0.41; MVPA vs. % of time in MVPA: r = 0.34	PA: – (Level 1) Very low SB: – (Level 2) Very low
Children’s ChronoType Questionnaire (CCTQ) - Japanese version [50]	*n* = 72 Age = 4.8 ± 0.8 years Sex = 51% girls	- Accelerometer (Actiwatch-2, non-dominant wrist, 60s epoch, threshold automatic sensitivity) - Sleep diary	Doubtful (both methods)	Accelerometer correlations (week/weekend): Sleep onset (r = 0.78/0.61), wake-up time (r = 0.82/0.71), midsleep point (r = 0.84/0.79), sleep period (r = 0.74/0.49), sleep latency (r = 0.51/0.38) Diary correlations (week/weekend): Bedtime (r = 0.77/0.64), get-up time (r = 0.83/0.71,) time in bed (r = 0.74/0.56)	Acc: + (Level 2) Very low Diary: + (Level 3) Very low
Physical activity and sedentary behavior questionnaire (based on Canadian Health Measures Survey) [64]^a^	*n* = 87 Age = 4–70 months Sex = 54% girls	Accelerometer (Actical, right hip, Wong/Adolph cut-points [78, 79])	Doubtful^g^ (both PA and SB)	Total PA vs. total PA min/day: MD = 131 min/day*, LoA = [− 80, 290], r = 0.39 (95% CI 0.19–0.56) Outdoor unstructured free play aside from school daycare setting vs. total PA min/day: r = 0.30 (95% CI 0.09–0.49) Unstructured play in school/daycare setting vs. total PA min/day: r = 0.42 (95% CI 0.23–0.58) Structured PA vs. total PA min/day: r = 0.26 (95% CI 0.05–0.46) Total SB: MD = 306 min/day*, LoA = [125, 460], r = 0.10 (95% CI − 0.12–0.33) Screen time: r = − 0.05 (95% CI − 0.27–0.18) Stroller time: r = 0.31 (95% CI 0.09–0.50) Motor vehicle time: r = − 0.09 (95% CI − 0.30–0.13)	PA: – (Level 1) Very low SB: – (Level 2) Very low
Sleep diary (nn) [46]	*n* = 52 Age = 13.7 ± 6.7 months (< 1 year old = 28, > 1 year old = 21) Sex = 56% girls	Accelerometer (Actigraph, Micro-mini RC, ankle, 60s epoch, Sadeh algorithm [80])	Doubtful	Sleep onset time (r = 0.89), sleep offset time (r = 0.91), nocturnal sleep duration (r = 0.75), the number of night awakenings (r = 0.46), WASO (r = 0.34)	+ (Level 2) Very low
Children’s Sleep Habits Questionnaire (CSHQ) [41]	*n* = 46 Age = 58 ± 10.25 months (range = 3–6 years) Sex = 50% girls	Accelerometer (Actiwatch 2, non-dominant wrist, 60s epoch, wake threshold value = 40)	Doubtful	Bedtime (week/weekend): r = 0.75/0.57, wake-up (week/weekend): r = 0.86/0.64, total sleep: r = − 0.08; Interval in hours/min (LoA): Bedtime (week/weekend) = ± 0:52 [− 1:31, 0:12]/± 1:20 [− 1:37, 1:02], wake-up (week/weekend) = ± 0:38 [− 0:45, 0:31]/± 0:32 [− 1:04, 2:00], total sleep = ± 2:39 [− 0:06, 5:12], WASO = ± 1:02 [− 2:17, − 0:14]	+ (Level 2) Very low
Own questionnaire (nn) [48]	*n* = 21 Age = 3.81 ± 0.28 years Sex = 43% girls	Accelerometer (Actiwatch, non-dominant ankle, 60s epochs, threshold automatic sensitivity)	Doubtful	Reported vs assumed sleeping hours (r = 0.90); Reported vs actual sleeping hours (r = 0.90); assumed vs actual sleeping hours (r = 0.99)	+ (Level 2) Very low
Sleep diary (nn) [47]	*n* = 80 Age = 25.34 ± 1.04 months Sex = 40% girls	Accelerometer (Mini-Mitter Actiwatch Actigraph, wrist/ankle, 60s epoch, high sensitivity automatic scoring algorithm/Sitnick “smooting” algorithm to detect night awakenings [81])	Doubtful	Sleep duration: r = 0.30, sleep efficiency: r = 0.02. Based on the a priori agreement criteria in this study, agreement was satisfactory for 70.0% of children with respect to sleep duration (≤90 min) and for 71.3% of children with respect to sleep efficiency (≤15%)	– (Level 2) Very low
Parent log (nn) [49]	*n* = 59 Age = 4.34 ± 0.75 years Sex = 53% girls	Accelerometer (Minimitter Motionlogger Actiwatch, non-dominant wrist, 60s epoch, Sadeh algorithm [80])	Doubtful	Weekday naps (t = 0.19), weekend naps (t = − 1.48), total napping (t = 0.004), weekday night sleep (t = − 5.33*), weekend night sleep (t = − 2.96*), total nights (t = − 5.01*), total sleep (t = − 5.07*)	– (Level 2) Very low
BISQ Spanisch version (BISQ-E) [35]	*n* = 27 Age = 3–30 months Sex = unknown	Sleep diary	Doubtful	Bedtime during weekdays (Mon-day through Friday) (r = 0.73), hours of night-time sleep (between 8 pm and 7 am) (r = 0.73), hours of daytime sleep (between 7 am and 8 pm) (r = 0.87) and number of night-time awakenings (r = 0.89), bedtime on weekend days (r = 0.69)	+ (Level 3) Very low
Sleep and Settle Questionnaire (SSQ) [45]	*n* = 34–36 mothers of infants Age = unknown Sex = unknown	Mothers attending a community class on sleep and settling difficulties with infants	Doubtful	Discriminant validity (mothers who report their babies sleep well vs. mothers attending a sleep and settle class): Time the infant sleeps (min): mornings (η^2^ = 0.03*), afternoons (NS), during night (η^2^ = 0.12*); How long to settle the baby to sleep (min): daytime (η^2^ = 0.17*), evening (η^2^ = 0.18*), night-time (η^2^ = 0.07*); Number of daytime sleeps (NS), number night awakenings (η^2^ = 0.17*).	+ (Level 3) Very low

*Abbreviations: Acc* accelerometer, *BISQ* Brief Infant Sleep Questionnaire, *CI* confidence interval, *COSMIN* COnsensus-based Standards for the selection of health Measurement Instruments, *CSHQ* Children’s Sleep Habits Questionnaire, *DLW* doubly labeled water, *h* hour, *ICC* intraclass correlation, *LoA* limits of agreement, *MD mean difference, min* minute, *MPA* moderate physical activity, *MVPA* moderate-to-vigorous physical activity, *nn* no name, *NS* not significant, *PA* physical activity, *PAEE* physical activity energy expenditure, *QA* Questionnaire, *r* correlation coefficient (Pearson or Spearman), *s* second, *SB* sedentary behavior, *ST* sedentary time, *VPA* vigorous physical activity, *Vid* videosomnography, *WASO* wake after sleep onset, * significant

^a^ Questionnaire included from previous review

^b^ Age presented as mean age ± SD (range)

^c^ Methodological quality based on the COSMIN risk of bias checklist

^d^ Result rating based on Table 1: + indicates ≥75% in accordance with hypotheses; ± indicates ≥50 to < 75% in accordance with hypotheses; − indicates < 50% accordance with hypotheses

^e^ Level of evidence based on criteria listed in Table 1

^f^ Quality of evidence grading based on the COSMIN guidelines

^g^ When multiple methods (e.g., cut-points) were used to analyze accelerometer data, the methodological quality rating was based on the method with the highest quality

### Description of questionnaires

Of the included questionnaires, 10 were designed for infants specifically (0–1 year old). These questionnaires all assessed constructs of sleep [37–39, 42–45]. Two questionnaires were designed for toddlers (1**–**3 years old), of which one assessed sleep [47], and one screen behavior [53]. Eleven questionnaires were designed for preschoolers (3**–**5 years old). Five of these questionnaires assessed constructs of sleep [40, 41, 48–50], two physical activity and sedentary behavior [58, 59], and four assessed constructs of all 24-h movement behaviors [62, 63, 66, 67]. Fourteen questionnaires were designed for multiple of the aforementioned age groups. Three questionnaires assessed sleep behavior in infants and toddlers [35, 36, 46]. Two questionnaires targeted both infants, toddlers, and preschoolers, of which one assessed screen behavior [52], and one physical activity and sedentary behavior [64]. Nine questionnaires targeted toddlers and preschoolers, of which one assessed sleep behavior [51], four assessed physical activity [54, 55], two assessed constructs of both physical activity and sedentary behavior [56, 57], and two assessed constructs of all 24-h movement behaviors [60, 61, 65].

Respondents of the questionnaires were parents or caregivers, except for two questionnaires that were completed by family child care providers, i.e., the modified Burdette proxy report and the modified Harro proxy report [55]. Recall periods varied across questionnaires, ranging from current day (*n* = 9) to a typical week (*n* = 3), with a typical (week or weekend) day being used most frequently (*n* = 13). Four questionnaires used ordinal response options (e.g., Likert scale), 17 continuous (e.g., duration in hours and/or minutes), one nominal and 14 questionnaires used a combination of these response options.

### Content validity

Six studies reported data on the comprehensiveness, comprehensibility and/or relevance of the items of the questionnaire under study (Table 3). Two of the examined questionnaires assessed constructs of sleep behavior, i.e., the Children Sleep Wake Scale (CSWS) [51] and Nepali version of the BISQ [36]. One questionnaire assessed screen behavior in children aged 0–5, i.e., the Technology Use Questionnaire (TechQ-U) [52]. The other three questionnaires, i.e., the Healthy Kids [60], the Family Health Survey [66], and the Surveillance of digital Media habits in early childhood Questionnaire (SMALLQ™) [65], assessed constructs of all 24-h movement behaviors in toddlers and/or preschoolers. Questionnaire development was reported in five studies [51, 52, 60, 65, 66]. These studies used cognitive interviews [60, 65, 66], semi-structured interviews [52], focus groups [65], and/or expert opinions [51, 52, 65] to evaluate content validity of the questionnaire. Two of these questionnaires were additionally pilot-tested in a small sample of caregivers to provide information on, for example, readability or time to complete the questionnaire [51, 65]. Overall, we graded the quality of evidence for the content validity of these questionnaires as low. Unfortunately, none of the questionnaires were evaluated on all aspects of content validity (i.e., relevance, comprehensiveness, and comprehensibility). For this reason, we could not rate the overall content validity of these questionnaires.

### Internal structure: structural validity, internal consistency, and cross-cultural validity

Internal consistency was evaluated for three questionnaires (Table 4), all assessing sleep behavior: the CSWS, Children’s Sleep Habits Questionnaire (CSHQ) and Children’s Sleep Habits Questionnaire for infants (CSQH-I) [39, 40, 51]. Internal consistency was evaluated by calculating Cronbach’s alpha of each subscale for all three questionnaires. The quality of evidence for the internal consistency was high for all three questionnaires. The internal consistency outcomes for the CSWS were rated as sufficient [51], whereas outcomes for the CSHQ and CSQH-I were rated as insufficient [39, 40]. The CSHQ and CSQH-I were also evaluated on structural validity by performing confirmatory factor analysis [40] and/or exploratory factor analysis [39, 40], both receiving a moderate evidence grading (Table 4). Structural validity outcomes of both questionnaires were considered sufficient. None of the translated or culturally adapted questionnaires were evaluated on cross-cultural validity or measurement invariance [35–37, 39, 40, 66].

### Reliability

Sixteen questionnaires were assessed on reliability (Table 5). Six of these questionnaires assessed constructs of sleep [35, 37, 39, 40, 45, 51], six assessed constructs of physical activity and/or sedentary behavior [52, 53, 56–59], and four assessed constructs of all 24-h movement behaviors [61–63, 67]. Reliability of nine questionnaires was evaluated by calculating ICC [40, 52, 53, 56–59, 63, 67], whereas Pearson or Spearman correlations were calculated for seven questionnaires [35, 37, 39, 45, 51, 61, 62]. Time interval between test and retest ranged between 7 days [53, 57] and 3 months [39]. Two questionnaires received a moderate quality of evidence grading for reliability, i.e., the Early Years Physical Activity Questionnaire (EY-PAQ) [57] and Preschool-age Children’s Physical Activity Questionnaire (Pre-PAQ) [58]. Reliability outcomes were considered insufficient for both questionnaires. Five questionnaires received a low quality of evidence grading, and nine questionnaires received a very low evidence grading.

### Measurement error

Measurement error was evaluated for one questionnaire, i.e., the Pre-PAQ [58]. The quality of evidence for the measurement error of this questionnaire was graded as moderate. This questionnaire demonstrated a measurement error ranging from 1.0**–**1.1 min for time spent in organized physical activities. Unfortunately, as the MIC for interpreting the measurement error outcomes was not defined, we could not give a final rating for measurement error. However, a measurement error of 1.0–1.1 min seems acceptable.

### Criterion and construct validity

Criterion validity was evaluated for one questionnaire (Table 6), i.e., the Children’s Physical Activity Questionnaire (CPAQ) [59]. The CPAQ was used to assess energy expenditure in 4- to 5-year-olds. This questionnaire received a low quality of evidence grading for criterion validity, and outcomes were considered insufficient.

Twenty-one studies evaluated the construct validity of a total of 26 different questionnaires (Table 6). Sixteen of these questionnaires assessed constructs of sleep [35, 38–50], eight assessed constructs of physical activity and/or sedentary behavior [54, 55, 57–59, 64], and two assessed constructs of all three movement behaviors [61, 62]. Thirteen studies evaluated construct validity using an objective comparator instrument (e.g., accelerometer) [41–43, 47–49, 55, 57–59, 62, 64], three used a subjective comparator instrument (e.g., diary) [35, 39, 61], and four studies used both [38, 44, 50, 54]. Nineteen studies used Pearson or Spearman correlations to evaluate construct validity, of which 10 studies also presented Bland-Altman plots [38, 41–44, 47, 54, 57–59, 64]. One study evaluated construct validity by comparing subgroups (i.e., discriminative validity) [45]. The quality of evidence for construct validity was graded as moderate for one questionnaire, i.e., the EY-PAQ [57], showing outcomes that were considered insufficient. Fifteen of the questionnaires received a low quality of evidence grading, and 10 questionnaires received a very low evidence grading.

## Discussion

This review summarizes studies that evaluated the measurement properties of proxy-report questionnaires for assessing physical activity, sedentary behavior, and/or sleep in 0- to 5-year-old children. The questionnaires varied in constructs, format, target population, and evaluated measurement properties. Unfortunately, while we identified 37 relevant questionnaires, none were considered valid and/or reliable for assessing one or more movement behaviors in children aged 0–5 years.

Proxy-report questionnaires for assessing all 24-h movement behaviors in this young age group are scarce. The majority of included questionnaires assessed one type of behavior, of which sleep behavior was assessed most frequently. Only six questionnaires assessed duration and/or frequency of all three behaviors (physical activity, sedentary behavior, and sleep) [60–63, 65, 67]. Unfortunately, these six questionnaires mostly used very few items per movement behavior, which questions the comprehensiveness of the items (e.g., a single item to assess sedentary behavior). Overall, 21 out of 37 questionnaires targeted children above 2 years old (e.g., preschoolers). Questionnaires that targeted solely infants all assessed sleep behavior (*n* = 10). Only one questionnaire (i.e., based on the Canadian Health Measures Survey) assessed physical activity and sedentary behavior in infants, next to toddlers and preschoolers (i.e., 0- to 6-year-old children) [64]. In addition, one questionnaire assessed screen use in 0- to 5-year-old children (i.e., TechU-Q) [52]. There were only two questionnaires that specifically targeted toddlers: a questionnaire assessing screen use (i.e., PREPS questionnaire) [53] and a sleep diary [47]. This further indicates the urgent need for developing questionnaires assessing 24-h movement behaviors in infants and toddlers. Notably is the large number of questionnaires (*n* = 14) that were designed for multiple age groups (e.g., toddlers and preschoolers). However, since infants, toddlers and preschoolers each have their own form and context of physical activity, sedentary behavior and sleep [9], tailored questionnaires are needed that fit the specific behavior of the target group.

There are two important issues that complicate drawing definite conclusions regarding the best available questionnaires. First, there is a lack of studies comprehensively evaluating measurement properties of proxy-report questionnaires. These findings are consistent with previous reviews [20, 21, 24]. For example, only one study reported information on the measurement error of a questionnaire [58]. Measurement error is an important characteristic of reliability, as it gives an indication of the systematic and random error expressed in units of measurement (e.g., minutes per day), which facilitates the interpretation of measurements [26]. Unfortunately, our systematic review confirms that measurement error is still largely underreported in studies evaluating measurement instruments [26]. Consequently, we are unable to correctly interpreted the results of research using these questionnaires to assess time spent in physical activity, sedentary behavior and/or sleep. Likewise, criterion validity was evaluated for only one questionnaire [59]. As in many of the included studies the comparison instrument was not considered a gold standard, these studies were considered to reflect construct validity. Additionally, none of the included studies evaluated the responsiveness of the questionnaire. However, for the purpose of longitudinally monitoring one or more movement behaviors, questionnaires should be able to detect changes over time [26, 30]. Furthermore, none of the translated or adapted questionnaires were evaluated on cross-cultural validity (measurement invariance). Without evaluating this type of validity, it remains unsure if the performance of the translated items are an adequate reflection of the performance of the items of the original questionnaire [30]. Moreover, there is a lack of studies describing the development or content validity of the questionnaire [20, 21]. Only six studies reported information that contributed to the content validity of the questionnaire [36, 51, 52, 60, 65, 66]. Consequently, it remains unclear if the respondents (e.g., parents, youth health care providers) consider the content of the questionnaire as relevant, comprehensive and comprehensible [32]. Without evaluating content validity, there is no certainty that the questionnaire measures what it intends to measure [26, 31]. It can be argued that it is difficult to proxy-report young children’s sporadic and intermittent behaviors [17], and therefore it would be unrealistic to expect that questionnaires can be used to assess 24-h movement behaviors very accurately in this target population. However, a more comprehensive development and evaluation of questionnaires (while including the target population and professionals in the process) would improve the quality of questionnaires. As appropriate measurement instruments are lacking, current evidence on young children’s 24-h movement behaviors and the effects on their growth and development is limited [82]. Therefore, improving the quality of questionnaires and alternative (device-based) measures would strongly benefit research in this field, and thereby future public health recommendations.

Second, the quality of evidence was graded as low or very low for the majority of evaluated measurement properties of included questionnaires (i.e., 40 out of 49 evaluated measurement properties across all questionnaires), which makes it even more difficult to draw conclusions about the most appropriate available questionnaires. Although there are a number of questionnaires of which reliability (test-retest reliability: *n* = 4, internal consistency: *n* = 1) and/or validity (construct validity: *n* = 11, structural validity: *n* = 2) outcomes were considered sufficient, only three of these questionnaires received at least a moderate quality of evidence grading for one of the evaluated measurement properties. This concerns the internal consistency of the CSWS [30] and the structural validity of the CSHQ [40] and CSHQ-I [39]. Unfortunately, other measurement properties of these questionnaires received a low or very low quality of evidence grading (e.g., reliability), or were not included in our review since these were not assessed in 0- to 5-year-old children (e.g., construct validity and structural validity of the CSWS [51]). Hence, no definite conclusions about the appropriateness of these questionnaires to assess sleep behavior in young children can be drawn.

There are a few reasons that contributed to the low quality of evidence of questionnaires. First, quality of evidence was often downgraded because of the small sample sizes (< 100 participants) included in studies (i.e., risk of imprecision) [30]. If multiple studies on the same measurement property of a questionnaire would have been available, pooling of study results would be possible, thereby solving this sample size issue [30].

Second, quality of evidence was often downgraded because of the doubtful methodology quality of studies (i.e., risk of bias) [30]. The lack of high-quality studies is consistent with findings from previous reviews by Hidding et al. [20, 21]. Common methodological limitations varied by evaluated measurement property. Concerning test-retest reliability, Pearson or Spearman correlations were often used without providing evidence that no systematic changes had occurred [30]. Furthermore, some reliability studies used inappropriate long time intervals between test and retest, e.g., 3 months, reducing the probability that children have remained stable in the interim period on the construct to be measured [30, 83]. The low methodological quality of construct validity studies was predominantly due to using comparator instruments with unknown or insufficient measurement properties. Most studies used, for example, non-validated diaries, or accelerometers without providing validated analyses methods (e.g., cut-points) in young children. Unfortunately, adequate comparator instruments with validated analyses methods are generally lacking in this young age group, making it difficult to ensure that comparator instruments measure the same constructs [13, 16, 29]. The doubtful methodological quality of content validity studies was predominantly due to the limited information reported on the methods used for the development of the questionnaire or the content validity evaluation. In particular, details on procedures of interviews or group meetings were lacking. For example, it was unclear whether interviewers were trained, whether data was coded independently, or whether data saturation was reached [31]. Moreover, not all aspects of content validity (i.e., relevance, comprehensiveness, and comprehensibility) were evaluated or the respondents (e.g., parents) were not included where appropriate.

It should be noted that some of the included questionnaires were not specifically aimed at assessing movement behaviors [53, 60, 61, 63, 65–67]. Consequently, frequency and/or duration of physical activity, sedentary behavior and/or sleep were only one of the many sub-constructs in these questionnaires. The Healthy Kids [60, 61], for example, is intended to assess obesity risk. This questionnaire included items related to children’s physical activity, sedentary behavior and sleep, as part of a larger questionnaire. Studies evaluating the content validity of such questionnaires considered the questionnaire as a whole, instead of each sub-construct separately [60, 65, 66]. Consequently, it was unclear whether all included items for physical activity, sedentary behavior and/or sleep were relevant and whether all key concepts were included (i.e., comprehensiveness). Future studies evaluating the content validity of questionnaires assessing multiple behaviors should evaluate each subscale or sub-construct separately to ensure an adequate evaluation of all included constructs [31].

### Strengths and limitations

A strength of this review is the standardized quality assessment of included studies, using the COSMIN guidelines [28, 30, 31]. Another strength is that we included questionnaires for assessing all 24-h movement behaviors (i.e., physical activity, sedentary behavior and sleep). There is growing evidence that all 24-h movement behaviors should be targeted when aiming for optimal health [5–10]. The current review gives a clear overview of the available proxy-report questionnaires to monitor these behaviors in young children. Additionally, screening, data extraction and methodological quality assessment have each been done by at least two independent researchers, minimizing the chance of bias. However, our review also has some limitations. We only included studies published in English, disregarding studies published in other languages. Consequently, our review might not be representative for questionnaires available in non-English speaking countries, although we only excluded a limited number of studies based on language. Furthermore, we rated the methodological quality of studies based on the information reported in each of the articles. When details on the methodology were lacking, studies received lower quality of evidence grades. We did not contact authors for additional information other than requesting the questionnaires that were used. Consequently, the quality of evidence of some studies might have been underestimated.

### Recommendations for future studies

Given the lack of questionnaires for assessing movement behaviors in young children, we recommend future studies to develop proxy-report questionnaires targeted specifically at children aged 0–5 years, including all 24-h movement behaviors. These questionnaires should preferably be tailored to fit the specific behavior of the subgroup (i.e., infants, toddlers or preschoolers), as movement behaviors quickly change during this period of rapid development.

When developing questionnaires, we strongly recommend future studies to evaluate the content validity of proxy-report questionnaires in a sample representing the target population (e.g., parents), including all components of content validity (i.e., relevance, comprehensiveness, and comprehensibility). We recommend these studies to use appropriate qualitative data collection methods, such as focus groups, interviews or concept mapping [31], and to evaluate the content validity of each subscale or sub-construct separately [31].

Next, high quality research on all other relevant measurement properties (both reliability and validity) of developed questionnaires is necessary. Future studies evaluating measurement properties of questionnaires are recommended to use standardized tools to increase study quality. We recommend the use of the COSMIN methodology for the design and reporting of these studies [84]. Specifically, high quality studies on measurement error, responsiveness, and cross-cultural validity of questionnaires are needed.

First, we recommend future studies that examine test-retest reliability to additionally report the measurement error, as both measurement properties can be calculated based on the same data and study design [84]. These studies should use at least two independent measurements with similar test conditions and an appropriate time interval, according to previous recommendations for youth physical activity questionnaires: > 1 day and < 1 week for questionnaires recalling the previous day, > 1 day and < 2 weeks for questionnaires recalling the recalling the previous week [19]. The preferred statistical method to assess the measurement error of continuous scores is the calculation of the standard error of measurement (SEM) [30]. For categorical scores we recommend calculating the percentage positive and negative agreement [84].

Second, more studies examining responsiveness of questionnaires are needed, reflecting longitudinal validity. Similar to construct validity, it is important to define hypotheses in advance when assessing responsiveness of a questionnaire, to enable drawing unbiased conclusions [84].

Third, we recommend future studies evaluating translated or culturally adapted questionnaires to examine cross-cultural validity. These studies are recommended to provide a clear description of the characteristics that should be similar in the different subgroups (e.g., demographics such as age) and provide clear information on the performance of the analysis (e.g. software program used, criteria for model fit) [84].

Last, as current studies evaluating the criterion and construct validity of questionnaires are limited due to a lack of comparators with sufficient measurement properties, we recommend future studies to improve comparator instruments and analysis methods to assess 24-h movement behaviors (e.g., accelerometer data analyses). Subsequently, improving the quality of questionnaires and alternative measurement methods would strongly benefit research in this field.

## Conclusion

None of the 37 proxy-report questionnaires included in this review were considered valid and/or reliable for assessing one or more movement behaviors in children aged 0–5 years. The lack of high-quality methodological studies that evaluate all relevant measurement properties of developed questionnaires hampers our ability to draw definite conclusions about the best available questionnaires. In addition, questionnaires for assessing 24-h movement behaviors in 0- to 5-year-olds are scarce. Thus, high-quality studies are required aimed to develop proxy-report questionnaires for this age group and to evaluate their measurement properties, starting with content validity. When sufficient content validity is established, the remaining relevant measurement properties (i.e., reliability, validity, responsiveness) should be evaluated [31, 84]. Until valid and reliable proxy-report questionnaires are available, caution is needed when interpreting results of research using proxy-reported physical activity, sedentary behavior and sleep in this young age group.

## Supplementary Information

**Additional file 1.**

**Additional file 2.**

## Data Availability

The data that support the findings of this review are available from the corresponding author upon reasonable request.
