# Supplementary material for: A systematic review of proxy-report questionnaires assessing physical activity, sedentary behavior and/or sleep in young children (aged 0–5 years)
Source: Int J Behav Nutr Phys Act. 2022 Feb 14;19:18. doi: 10.1186/s12966-022-01251-x (PMC8845346; doi:10.1186/s12966-022-01251-x)
Supplement: Supplementary file 1 — Additional file 1. [file 12966_2022_1251_MOESM1_ESM.pdf]

## Additional file 1: Search strategy

This additional file contains the search terms used to conduct the search across three electronic databases: PubMed, Embase, and SPORTDiscus.

## Search strategy – Physical Activity and Sedentary Behavior

**PubMed**

<u>Population</u>

"Infant"[Mesh:noexp] OR "Infant, Newborn"[Mesh:noexp] OR "Child, Preschool"[Mesh] OR infan*[tiab] OR newborn*[tiab] OR new-born*[tiab] OR neonate*[tiab] OR baby[tiab] OR babies[tiab] OR toddler*[tiab] OR preschool*[tiab] OR pre-school*[tiab] OR kindergarten*[tiab] OR childcare*[tiab] OR daycare*[tiab] OR nurser*[tiab] OR ECEC[tiab] OR early childhood[tiab] OR early years[tiab] OR early life[tiab] OR children[tiab]

AND

<u>24h Movement behavior - Physical activity</u>

"Motor Activity"[Mesh:NoExp] OR "Exercise"[Mesh] OR "Sports"[Mesh] OR motor activit*[tiab] OR physical activit*[tiab] OR locomotor activit*[tiab] OR exercis*[tiab] OR running[tiab] OR run[tiab] or sport*[tiab] OR active[tiab] OR walk*[tiab] OR outdoor[tiab] OR play*[tiab] OR bicycle[tiab] OR cycle[tiab] OR bicycling[tiab] OR cycling[tiab] OR biking[tiab] OR "tummy time"[tiab] OR "floor time"[tiab] OR "prone position"[tiab] OR crawl*[tiab] OR swim*[tiab] OR "rough and tumble"[tiab] OR "Movement"[MeSH:NoExp] OR movement[tiab]

OR

<u>24h Movement behaviour - Sedentary Behavior and Screen Time</u>

"Sedentary Behavior"[Mesh:NoExp] OR sedentary[tiab] OR stationary behavio*[tiab] OR physically inactive[tiab] OR physical inactiv*[tiab] OR sitting[tiab] OR computer time[tiab] OR computer use[tiab] OR screen time[tiab] OR television[tiab] OR "TV time"[tiab] OR "TV viewing"[tiab] OR "watching TV"[tiab] OR videogam*[tiab] OR gaming[tiab] OR tablet use[tiab] OR "tablet time"[tiab] OR screen entertainment[tiab] OR screen based[tiab]

AND

<u>Measurement properties Proxy-report measures</u>

instrumentation[sh] OR methods[sh] OR Validation Studies[pt] OR Comparative Study[pt] OR "psychometrics"[MeSH] OR psychometr*[tiab] OR clinimetr*[tw] OR clinometr*[tw] OR "outcome assessment (health care)"[MeSH] OR outcome assessment[tiab] OR outcome measure*[tw] OR "observer variation"[MeSH] OR observer variation[tiab] OR "Health Status Indicators"[Mesh] OR "reproducibility of results"[MeSH] OR reproducib*[tiab] OR "discriminant analysis"[MeSH] OR reliab*[tiab] OR unreliab*[tiab] OR valid*[tiab] OR coefficient[tiab] OR homogeneity[tiab] OR homogeneous[tiab] OR "internal consistency"[tiab] OR (cronbach*[tiab] AND (alpha[tiab] OR alphas[tiab])) OR (item[tiab] AND (correlation*[tiab] OR selection*[tiab] OR reduction*[tiab])) OR agreement[tw] OR precision[tw] OR imprecision[tw] OR "precise values"[tw] OR test-retest[tiab] OR (test[tiab] AND retest[tiab]) OR (reliab*[tiab] AND (test[tiab] OR retest[tiab])) OR stability[tiab] OR interrater[tiab] OR inter-rater[tiab] OR intrarater[tiab] OR intra-rater[tiab] OR intertester[tiab] OR inter-tester[tiab] OR intratester[tiab] OR intra-tester[tiab] OR interobserver[tiab] OR inter-observer[tiab] OR intraobserver[tiab] OR intraobserver[tiab] OR intertechnician[tiab] OR inter-technician[tiab] OR intratechnician[tiab] OR intratechnician[tiab] OR interexaminer[tiab] OR inter-examiner[tiab] OR intraexaminer[tiab] OR intraexaminer[tiab] OR interassay[tiab] OR inter-assay[tiab] OR intraassay[tiab] OR intra-assay[tiab] OR interindividual[tiab] OR inter-individual[tiab] OR intraindividual[tiab] OR intra-individual[tiab] OR interparticipant[tiab] OR inter-participant[tiab] OR intraparticipant[tiab] OR intra-participant[tiab] OR kappa[tiab] OR kappa's[tiab] OR kappas[tiab] OR repeatab*[tw] OR ((replicab*[tw] OR

repeated[tw]) AND (measure[tw] OR measures[tw] OR findings[tw] OR result[tw] OR results[tw] OR test[tw] OR tests[tw])) OR generaliza*[tiab] OR generalisa*[tiab] OR concordance[tiab] OR (intraclass[tiab] AND correlation*[tiab]) OR discriminative[tiab] OR "known group"[tiab] OR factor analysis[tiab] OR factor analyses[tiab] OR dimension*[tiab] OR subscale*[tiab] OR (multitrait[tiab] AND scaling[tiab] AND (analysis[tiab] OR analyses[tiab])) OR item discriminant[tiab] OR interscale correlation*[tiab] OR error[tiab] OR errors[tiab] OR "individual variability"[tiab] OR (variability[tiab] AND (analysis[tiab] OR values[tiab])) OR (uncertainty[tiab] AND (measurement[tiab] OR measuring[tiab])) OR "standard error of measurement"[tiab] OR sensitiv*[tiab] OR responsive*[tiab] OR ((minimal[tiab] OR minimally[tiab] OR clinical[tiab] OR clinically[tiab]) AND (important[tiab] OR significant[tiab] OR detectable[tiab]) AND (change[tiab] OR difference[tiab])) OR (small*[tiab] AND (real[tiab] OR detectable[tiab]) AND (change[tiab] OR difference[tiab])) OR meaningful change[tiab] OR "ceiling effect"[tiab] OR "floor effect"[tiab] OR "Item response model"[tiab] OR IRT[tiab] OR Rasch[tiab] OR "Differential item functioning"[tiab] OR DIF[tiab] OR "computer adaptive testing"[tiab] OR "item bank"[tiab] OR "cross cultural equivalence"[tiab]

AND

(("Parents"[Mesh] OR parent[tiab] OR "Proxy"[Mesh] OR proxy[tiab] OR "Caregivers"[Mesh] OR carer[tiab] OR caregiver[tiab]) AND (report[tiab] OR reported[tiab] OR reporting[tiab] OR rated[tiab] OR rating[tiab] OR ratings[tiab] OR assessed[tiab] OR assessment[tiab] OR assessments[tiab])) OR "Surveys and Questionnaires"[Mesh] OR questionnaire*[tiab] OR instrument*[tiab] OR survey*[tiab] OR scal*[tiab]

NOT

Potential 'NOT' terms

("Diseases Category"[Majr] OR "Attention Deficit and Disruptive Behavior Disorders"[Majr] OR "Child Development Disorders, Pervasive"[Majr]) OR ("Animals"[Mesh] NOT "Humans"[Mesh])

**Embase**

Population

'infant'/de OR 'baby'/exp OR 'newborn'/exp OR 'preschool child'/exp OR 'toddler'/exp OR 'early childhood'/exp OR 'early life'/exp OR infan*:ab,ti,kw OR newborn*:ab,ti,kw OR 'new-born*':ab,ti,kw OR neonate*:ab,ti,kw OR baby:ab,ti,kw OR babies:ab,ti,kw OR toddler*:ab,ti,kw OR preschool*:ab,ti,kw OR 'pre-school*':ab,ti,kw OR kindergarten*:ab,ti,kw OR childcare*:ab,ti,kw OR daycare*:ab,ti,kw OR nurser*:ab,ti,kw OR ECEC:ab,ti,kw OR 'early childhood':ab,ti,kw OR 'early years':ab,ti,kw OR 'early life':ab,ti,kw OR children:ab,ti,kw

AND

24h Movement behavior - Physical activity

'motor activity'/exp OR 'exercise'/exp OR 'sport'/exp OR 'motor activit*':ab,ti,kw OR 'physical activit*':ab,ti,kw OR 'locomotor activit*':ab,ti,kw OR 'exercis*':ab,ti,kw OR 'running':ab,ti,kw OR 'run':ab,ti,kw OR 'sport*':ab,ti,kw OR 'active':ab,ti,kw OR 'walk*':ab,ti,kw OR 'outdoor':ab,ti,kw OR 'play*':ab,ti,kw OR 'bicycle':ab,ti,kw OR 'cycle':ab,ti,kw OR 'bicycling':ab,ti,kw OR 'cycling':ab,ti,kw OR 'biking':ab,ti,kw OR 'tummy time':ab,ti,kw OR 'floor time':ab,ti,kw OR 'prone position':ab,ti,kw OR 'crawl*':ab,ti,kw OR 'swim*':ab,ti,kw OR 'rough and tumble':ab,ti,kw OR 'movement (physiology)'/exp OR 'movement':ab,ti,kw

OR

24h Movement behavior - Sedentary Behavior and Screen Time

'sedentary lifestyle'/exp OR 'sedentary':ab,ti,kw OR 'stationary behavio*':ab,ti,kw OR 'physically inactive':ab,ti,kw OR 'physical inactiv*':ab,ti,kw OR 'sitting'/exp OR 'sitting':ab,ti,kw OR 'computer time':ab,ti,kw OR 'computer use':ab,ti,kw OR 'screen time':ab,ti,kw OR 'television':ab,ti,kw OR 'TV time':ab,ti,kw OR 'TV viewing':ab,ti,kw OR 'watching TV':ab,ti,kw OR 'videogam*':ab,ti,kw OR 'gaming':ab,ti,kw OR 'tablet use':ab,ti,kw OR 'tablet time':ab,ti,kw OR 'screen entertainment':ab,ti,kw OR 'screen based':ab,ti,kw

AND

Measurement properties Proxy-report measures

'intermethod comparison'/exp OR 'data collection method'/exp OR 'validation study'/exp OR 'feasibility study'/exp OR 'pilot study'/exp OR 'psychometry'/exp OR 'reproducibility'/exp OR reproducib*:ab,ti,kw OR'audit':ab,ti,kw OR psychometr*:ab,ti,kw OR clinimetr*:ab,ti,kw OR clinometr*:ab,ti,kw OR 'observer variation'/exp OR 'observer variation':ab,ti,kw OR 'discriminant analysis'/exp OR 'validity'/exp OR reliab*:ab,ti,kw OR valid*:ab,ti,kw OR 'coefficient':ab,ti,kw OR 'internal consistency':ab,ti,kw OR (cronbach*:ab,ti,kw AND ('alpha':ab,ti,kw OR 'alphas':ab,ti,kw)) OR 'item correlation':ab,ti,kw OR 'item correlations':ab,ti,kw OR 'item selection':ab,ti,kw OR 'item selections':ab,ti,kw OR 'item reduction':ab,ti,kw OR 'item reductions':ab,ti,kw OR 'agreement':ab,ti,kw OR 'precision':ab,ti,kw OR 'imprecision':ab,ti,kw OR 'precise values':ab,ti,kw OR 'test-retest':ab,ti,kw OR ('test':ab,ti,kw AND 'retest':ab,ti,kw) OR (reliab*:ab,ti,kw AND ('test':ab,ti,kw OR 'retest':ab,ti,kw)) OR 'stability':ab,ti,kw OR 'interrater':ab,ti,kw OR 'inter-rater':ab,ti,kw OR 'intrarater':ab,ti,kw OR 'intra-rater':ab,ti,kw OR 'intertester':ab,ti,kw OR 'inter-tester':ab,ti,kw OR 'intratester':ab,ti,kw OR 'intra- tester':ab,ti,kw OR 'interobeserver':ab,ti,kw OR 'inter-observer':ab,ti,kw OR 'intraobserver':ab,ti,kw OR 'intra- observer':ab,ti,kw OR 'intertechnician':ab,ti,kw OR 'inter-technician':ab,ti,kw OR 'intratechnician':ab,ti,kw OR 'intra- technician':ab,ti,kw OR 'interexaminer':ab,ti,kw OR 'inter-examiner':ab,ti,kw OR 'intraexaminer':ab,ti,kw OR 'intra- examiner':ab,ti,kw OR 'interassay':ab,ti,kw OR 'inter-assay':ab,ti,kw OR 'intraassay':ab,ti,kw OR 'intra-assay':ab,ti,kw OR 'interindividual':ab,ti,kw OR 'inter-individual':ab,ti,kw OR 'intraindividual':ab,ti,kw OR 'intra-individual':ab,ti,kw OR 'interparticipant':ab,ti,kw OR 'inter-participant':ab,ti,kw OR 'intraparticipant':ab,ti,kw OR 'intra- participant':ab,ti,kw OR 'kappa':ab,ti,kw OR 'kappas':ab,ti,kw OR 'coefficient of variation':ab,ti,kw OR repeatab*:ab,ti,kw OR (replicab*:ab,ti,kw OR 'repeated':ab,ti,kw AND ('measure':ab,ti,kw OR 'measures':ab,ti,kw OR 'findings':ab,ti,kw OR 'result':ab,ti,kw OR 'results':ab,ti,kw OR 'test':ab,ti,kw OR 'tests':ab,ti,kw)) OR generaliza*:ab,ti,kw OR generalisa*:ab,ti,kw OR 'concordance':ab,ti,kw OR ('intraclass':ab,ti,kw AND correlation*:ab,ti,kw) OR 'discriminative':ab,ti,kw OR 'known group':ab,ti,kw OR 'factor analysis':ab,ti,kw OR 'factor analyses':ab,ti,kw OR 'factor structure':ab,ti,kw OR 'factor structures':ab,ti,kw OR 'dimensionality':ab,ti,kw OR subscale*:ab,ti,kw OR 'multitrait scaling analysis':ab,ti,kw OR 'multitrait scaling analyses':ab,ti,kw OR 'item discriminant':ab,ti,kw OR 'interscale correlation':ab,ti,kw OR 'interscale correlations':ab,ti,kw OR ('error':ab,ti,kw OR 'errors':ab,ti,kw AND (measure*:ab,ti,kw OR correlat*:ab,ti,kw OR evaluat*:ab,ti,kw OR 'accuracy':ab,ti,kw OR 'accurate':ab,ti,kw OR 'precision':ab,ti,kw OR 'mean':ab,ti,kw)) OR 'individual variability':ab,ti,kw OR 'interval variability':ab,ti,kw OR 'rate variability':ab,ti,kw OR 'variability analysis':ab,ti,kw OR ('uncertainty':ab,ti,kw AND ('measurement':ab,ti,kw OR 'measuring':ab,ti,kw)) OR 'standard error of measurement':ab,ti,kw OR sensitiv*:ab,ti,kw OR responsive*:ab,ti,kw OR ('limit':ab,ti,kw AND 'detection':ab,ti,kw) OR 'minimal detectable concentration':ab,ti,kw OR interpretab*:ab,ti,kw OR (small*:ab,ti,kw AND ('real':ab,ti,kw OR 'detectable':ab,ti,kw) AND ('change':ab,ti,kw OR 'difference':ab,ti,kw)) OR 'meaningful change':ab,ti,kw OR 'minimal important change':ab,ti,kw OR 'minimal important difference':ab,ti,kw OR 'minimally important change':ab,ti,kw OR 'minimally important difference':ab,ti,kw OR 'minimal detectable change':ab,ti,kw OR 'minimal detectable difference':ab,ti,kw OR 'minimally detectable change':ab,ti,kw OR 'minimally detectable difference':ab,ti,kw OR 'minimal real change':ab,ti,kw OR 'minimal real difference':ab,ti,kw OR 'minimally real change':ab,ti,kw OR 'minimally real difference':ab,ti,kw OR 'ceiling effect':ab,ti,kw OR 'floor effect':ab,ti,kw OR 'item response model':ab,ti,kw OR 'irt':ab,ti,kw OR 'rasch':ab,ti,kw OR 'differential item functioning':ab,ti,kw OR 'dif':ab,ti,kw OR 'computer adaptive testing':ab,ti,kw OR 'item bank':ab,ti,kw OR 'cross-cultural equivalence':ab,ti,kw

AND

(('parent'/exp OR 'parent':ab,ti,kw OR 'proxy'/exp OR 'proxy':ab,ti,kw OR 'carer'/exp OR 'carer':ab,ti,kw OR 'caregiver'/exp OR 'caregiver':ab,ti,kw) AND ('report':ab,ti,kw OR 'reported':ab,ti,kw OR 'reporting':ab,ti,kw OR 'rated':ab,ti,kw OR 'rating':ab,ti,kw OR 'ratings':ab,ti,kw OR 'assessed':ab,ti,kw OR 'assessment':ab,ti,kw OR 'assessments':ab,ti,kw)) OR 'questionnaire'/exp OR 'questionnair*':ab,ti,kw OR 'instrument*':ab,ti,kw OR 'survey*':ab,ti,kw OR 'scal*':ab,ti,kw

NOT

Potential ‘NOT’ terms

NOT ('conference abstract'/it OR 'conference review'/it)
NOT ([animals]/lim NOT [humans]/lim)
NOT ('diseases'/exp/mj OR 'attention deficit disorder'/exp/mj OR 'autism'/exp/mj)

**SPORTDiscus**

<u>Population</u>

TI(infan* OR newborn* OR "new-born*" OR neonate* OR baby OR babies OR toddler* OR preschool* OR "pre-school*" OR kindergarten* OR childcare* OR daycare* OR nurser* OR ECEC OR "early childhood" OR "early years" OR "early life" OR children) OR AB(infan* OR newborn* OR "new-born*" OR neonate* OR baby OR babies OR toddler* OR preschool* OR "pre-school*" OR kindergarten* OR childcare* OR daycare* OR nurser* OR ECEC OR "early childhood" OR "early years" OR "early life" OR children) OR KW(infan* OR newborn* OR "new-born*" OR neonate* OR baby OR babies OR toddler* OR preschool* OR "pre-school*" OR kindergarten* OR childcare* OR daycare* OR nurser* OR ECEC OR "early childhood" OR "early years" OR "early life" OR children)

AND

<u>24h Movement behavior - Physical activity</u>

DE("HUMAN locomotion" OR "RUNNING" OR "SWIMMING" OR "WALKING" OR "CYCLING") OR SU("PHYSICAL activity" OR "BODY movement" OR "PHYSICAL fitness" OR "EXERCISE") OR TI("motor activit*" OR "physical activit*" OR "locomotor activit*" OR exercis* OR running OR run OR sport* OR active OR walk* OR outdoor OR play* OR bicycle OR cycle OR bicycling OR cycling OR biking OR "tummy time" OR "floor time" OR "prone position" OR crawl* OR swim* OR "rough and tumble" OR movement) OR AB("motor activit*" OR "physical activit*" OR "locomotor activit*" OR exercis* OR running OR run OR sport* OR active OR walk* OR outdoor OR play* OR bicycle OR cycle OR bicycling OR cycling OR biking OR "tummy time" OR "floor time" OR "prone position" OR crawl*OR swim*OR "rough and tumble" OR movement) OR KW("motor activit*" OR "physical activit*" OR "locomotor activit*" OR exercis* OR running OR run OR sport* OR active OR walk* OR outdoor OR play* OR bicycle OR cycle OR bicycling OR cycling OR biking OR "tummy time" OR "floor time" OR "prone position" OR crawl* OR swim* OR "rough and tumble" OR movement)

OR

<u>24h Movement behavior - Sedentary Behavior and Screen Time</u>

SU("SEDENTARY behavior" OR "SEDENTARY behavior in children" OR "SEDENTARY lifestyles" OR "SEDENTARY people") OR TI(sedentary OR "physical inactiv*" OR "physically inactive" OR "stationary behavio*" OR sitting OR "computer time" OR "computer use" OR "screen time" OR television OR "TV time" OR "TV viewing" OR "watching TV" OR videogam* OR gaming OR "tablet use" OR "tablet time" OR "screen entertainment" OR "screen based") OR AB(sedentary OR "physical inactiv*" OR "physically inactive" OR "stationary behavio*" OR sitting OR "computer time" OR "computer use" OR "screen time" OR television OR "TV time" OR "TV viewing" OR "watching TV" OR videogam* OR gaming OR "tablet use" OR "tablet time" OR "screen entertainment" OR "screen based") OR KW(sedentary OR "physical inactiv*" OR "physically inactive" OR "stationary behavio*" OR sitting OR "computer time" OR "computer use" OR "screen time" OR television OR "TV time" OR "TV viewing" OR "watching TV" OR videogam* OR gaming OR "tablet use" OR "tablet time" OR "screen entertainment" OR "screen based")

AND

<u>Measurement properties Proxy-report measures</u>

SU(QUESTIONNAIR* OR SURVEY*) OR (DE ("CAREGIVERS") AND TI(report OR reported OR reporting OR rated OR rating OR ratings OR assessed OR assessment OR assessments)) OR (DE ("CAREGIVERS") AND AB(report OR reported OR reporting OR rated OR rating OR ratings OR assessed OR assessment OR assessments)) OR (DE ("CAREGIVERS") AND KW(report OR reported OR reporting OR rated OR rating OR ratings OR assessed OR assessment OR assessments)) OR TI(((parent OR proxy OR carer) AND (report OR reported OR reporting OR rated OR rating OR ratings OR assessed OR assessment OR assessments)) OR

questionnair* OR instrument* OR survey* OR scal*) OR AB(((parent OR proxy OR carer) AND (report OR reported OR reporting OR rated OR rating OR ratings OR assessed OR assessment OR assessments)) OR questionnair* OR instrument* OR survey* OR scal*) OR KW(((parent OR proxy OR carer) AND (report OR reported OR reporting OR rated OR rating OR ratings OR assessed OR assessment OR assessments)) OR questionnair* OR instrument* OR survey* OR scal*)

NOT

<u>Potential 'NOT' terms</u>

(MM "CEREBRAL palsy" OR MM "ATTENTION-deficit hyperactivity disorder" OR MM "ATTENTION-deficit-disordered children" OR TI("cerebral palsy" OR autism OR autistic OR "attention deficit" OR ADHD) OR KW("cerebral palsy" OR autism OR autistic OR "attention deficit" OR ADHD))

## Search strategy – Sleep

**PubMed**

<u>Population</u>

"Infant"[Mesh:noexp] OR "Infant, Newborn"[Mesh:noexp] OR "Child, Preschool"[Mesh] OR infan*[tiab] OR newborn*[tiab] OR new-born*[tiab] OR neonate*[tiab] OR baby[tiab] OR babies[tiab] OR toddler*[tiab] OR preschool*[tiab] OR pre-school*[tiab] OR kindergarten*[tiab] OR childcare*[tiab] OR daycare*[tiab] OR nurser*[tiab] OR ECEC[tiab] OR early childhood[tiab] OR early years[tiab] OR early life[tiab] OR children[tiab]

AND

<u>24h Movement behavior – Sleep</u>

"Sleep"[Mesh:NoExp] OR sleep*[tiab] OR time in bed[tiab] OR bed time[tiab] OR bedtime[tiab] OR night rest[tiab] OR night awakening*[tiab] OR night waking*[tiab] OR night wakening*[tiab] OR nap[tiab] OR napping[tiab] OR asleep[tiab]

AND

<u>Measurement properties Proxy-report measures</u>

instrumentation[sh] OR methods[sh] OR Validation Studies[pt] OR Comparative Study[pt] OR "psychometrics"[MeSH] OR psychometr*[tiab] OR clinimetr*[tw] OR clinometr*[tw] OR "outcome assessment (health care)"[MeSH] OR outcome assessment[tiab] OR outcome measure*[tw] OR "observer variation"[MeSH] OR observer variation[tiab] OR "Health Status Indicators"[Mesh] OR "reproducibility of results"[MeSH] OR reproducib*[tiab] OR "discriminant analysis"[MeSH] OR reliab*[tiab] OR unreliab*[tiab] OR valid*[tiab] OR coefficient[tiab] OR homogeneity[tiab] OR homogeneous[tiab] OR "internal consistency"[tiab] OR (cronbach*[tiab] AND (alpha[tiab] OR alphas[tiab])) OR (item[tiab] AND (correlation*[tiab] OR selection*[tiab] OR reduction*[tiab])) OR agreement[tw] OR precision[tw] OR imprecision[tw] OR "precise values"[tw] OR test-retest[tiab] OR (test[tiab] AND retest[tiab]) OR (reliab*[tiab] AND (test[tiab] OR retest[tiab])) OR stability[tiab] OR interrater[tiab] OR inter-rater[tiab] OR intrarater[tiab] OR intra-rater[tiab] OR intertester[tiab] OR inter-tester[tiab] OR intratester[tiab] OR intra-tester[tiab] OR interobserver[tiab] OR inter-observer[tiab] OR intraobserver[tiab] OR intraobserver[tiab] OR intertechnician[tiab] OR inter-technician[tiab] OR intratechnician[tiab] OR intratechnician[tiab] OR interexaminer[tiab] OR inter-examiner[tiab] OR intraexaminer[tiab] OR intraexaminer[tiab] OR interassay[tiab] OR inter-assay[tiab] OR intraassay[tiab] OR intra-assay[tiab] OR interindividual[tiab] OR inter-individual[tiab] OR intraindividual[tiab] OR intra-individual[tiab] OR interparticipant[tiab] OR inter-participant[tiab] OR intraparticipant[tiab] OR intra-participant[tiab] OR kappa[tiab] OR kappa's[tiab] OR kappas[tiab] OR repeatab*[tw] OR ((replicab*[tw] OR repeated[tw]) AND (measure[tw] OR measures[tw] OR findings[tw] OR result[tw] OR results[tw] OR test[tw] OR tests[tw])) OR generaliza*[tiab] OR generalisa*[tiab] OR concordance[tiab] OR (intraclass[tiab] AND correlation*[tiab]) OR discriminative[tiab] OR "known group"[tiab] OR factor analysis[tiab] OR factor analyses[tiab] OR dimension*[tiab] OR subscale*[tiab] OR (multitrait[tiab] AND scaling[tiab] AND

(analysis[tiab] OR analyses[tiab])) OR item discriminant[tiab] OR interscale correlation*[tiab] OR error[tiab] OR errors[tiab] OR "individual variability"[tiab] OR (variability[tiab] AND (analysis[tiab] OR values[tiab])) OR (uncertainty[tiab] AND (measurement[tiab] OR measuring[tiab])) OR "standard error of measurement"[tiab] OR sensitiv*[tiab] OR responsive*[tiab] OR ((minimal[tiab] OR minimally[tiab] OR clinical[tiab] OR clinically[tiab]) AND (important[tiab] OR significant[tiab] OR detectable[tiab]) AND (change[tiab] OR difference[tiab])) OR (small*[tiab] AND (real[tiab] OR detectable[tiab]) AND (change[tiab] OR difference[tiab])) OR meaningful change[tiab] OR "ceiling effect"[tiab] OR "floor effect"[tiab] OR "Item response model"[tiab] OR IRT[tiab] OR Rasch[tiab] OR "Differential item functioning"[tiab] OR DIF[tiab] OR "computer adaptive testing"[tiab] OR "item bank"[tiab] OR "cross cultural equivalence"[tiab]

AND

(("Parents"[Mesh] OR parent[tiab] OR "Proxy"[Mesh] OR proxy[tiab] OR "Caregivers"[Mesh] OR carer[tiab] OR caregiver[tiab]) AND (report[tiab] OR reported[tiab] OR reporting[tiab] OR rated[tiab] OR rating[tiab] OR ratings[tiab] OR assessed[tiab] OR assessment[tiab] OR assessments[tiab])) OR "Surveys and Questionnaires"[Mesh] OR questionnaire*[tiab] OR instrument*[tiab] OR survey*[tiab] OR scal*[tiab]

NOT

<u>Potential ‘NOT’ terms</u>

("Diseases Category"[Majr] OR "Attention Deficit and Disruptive Behavior Disorders"[Majr] OR "Child Development Disorders, Pervasive"[Majr]) OR ("Animals"[Mesh] NOT "Humans"[Mesh])

**Embase**

<u>Population</u>

'infant'/de OR 'baby'/exp OR 'newborn'/exp OR 'preschool child'/exp OR 'toddler'/exp OR 'early childhood'/exp OR 'early life'/exp OR infan*:ab,ti,kw OR newborn*:ab,ti,kw OR 'new-born*':ab,ti,kw OR neonate*:ab,ti,kw OR baby:ab,ti,kw OR babies:ab,ti,kw OR toddler*:ab,ti,kw OR preschool*:ab,ti,kw OR 'pre-school*':ab,ti,kw OR kindergarten*:ab,ti,kw OR childcare*:ab,ti,kw OR daycare*:ab,ti,kw OR nurser*:ab,ti,kw OR ECEC:ab,ti,kw OR 'early childhood':ab,ti,kw OR 'early years':ab,ti,kw OR 'early life':ab,ti,kw OR children:ab,ti,kw

AND

<u>24h Movement behavior – Sleep</u>

'Sleep'/exp OR 'sleep*':ab,ti,kw OR 'time in bed':ab,ti,kw OR 'bed time':ab,ti,kw OR 'bedtime':ab,ti,kw OR 'night rest':ab,ti,kw OR 'night awakening':ab,ti,kw OR 'night waking':ab,ti,kw OR 'night wakening*':ab,ti,kw OR 'nap':ab,ti,kw OR 'napping':ab,ti,kw OR 'asleep':ab,ti,kw

AND

<u>Measurement properties Proxy-report measures</u>

'intermethod comparison'/exp OR 'data collection method'/exp OR 'validation study'/exp OR 'feasibility study'/exp OR 'pilot study'/exp OR 'psychometry'/exp OR 'reproducibility'/exp OR reproducib*:ab,ti,kw OR'audit':ab,ti,kw OR psychometr*:ab,ti,kw OR clinimetr*:ab,ti,kw OR clinometr*:ab,ti,kw OR 'observer variation'/exp OR 'observer variation':ab,ti,kw OR 'discriminant analysis'/exp OR 'validity'/exp OR reliab*:ab,ti,kw OR valid*:ab,ti,kw OR 'coefficient':ab,ti,kw OR 'internal consistency':ab,ti,kw OR (cronbach*:ab,ti,kw AND ('alpha':ab,ti,kw OR 'alphas':ab,ti,kw)) OR 'item correlation':ab,ti,kw OR 'item correlations':ab,ti,kw OR 'item selection':ab,ti,kw OR 'item selections':ab,ti,kw OR 'item reduction':ab,ti,kw OR 'item reductions':ab,ti,kw OR 'agreement':ab,ti,kw OR 'precision':ab,ti,kw OR 'imprecision':ab,ti,kw OR 'precise values':ab,ti,kw OR 'test-retest':ab,ti,kw OR ('test':ab,ti,kw AND 'retest':ab,ti,kw) OR (reliab*:ab,ti,kw AND ('test':ab,ti,kw OR 'retest':ab,ti,kw)) OR 'stability':ab,ti,kw OR 'interrater':ab,ti,kw OR 'inter-rater':ab,ti,kw OR 'intrarater':ab,ti,kw OR 'intra-rater':ab,ti,kw OR 'intertester':ab,ti,kw OR 'inter-tester':ab,ti,kw OR 'intratester':ab,ti,kw OR 'intra- tester':ab,ti,kw OR 'interobeserver':ab,ti,kw OR 'inter-observer':ab,ti,kw OR 'intraobserver':ab,ti,kw OR 'intra- observer':ab,ti,kw OR 'intertechnician':ab,ti,kw OR 'inter-technician':ab,ti,kw OR 'intratechnician':ab,ti,kw OR 'intra- technician':ab,ti,kw OR 'interexaminer':ab,ti,kw OR 'inter-examiner':ab,ti,kw OR 'intraexaminer':ab,ti,kw OR 'intra- examiner':ab,ti,kw OR 'interassay':ab,ti,kw OR 'inter-

assay':ab,ti,kw OR 'intraassay':ab,ti,kw OR 'intra-assay':ab,ti,kw OR 'interindividual':ab,ti,kw OR 'inter-individual':ab,ti,kw OR 'intraindividual':ab,ti,kw OR 'intra-individual':ab,ti,kw OR 'interparticipant':ab,ti,kw OR 'inter-participant':ab,ti,kw OR 'intraparticipant':ab,ti,kw OR 'intra- participant':ab,ti,kw OR 'kappa':ab,ti,kw OR 'kappas':ab,ti,kw OR 'coefficient of variation':ab,ti,kw OR repeatab*:ab,ti,kw OR (replicab*:ab,ti,kw OR 'repeated':ab,ti,kw AND ('measure':ab,ti,kw OR 'measures':ab,ti,kw OR 'findings':ab,ti,kw OR 'result':ab,ti,kw OR 'results':ab,ti,kw OR 'test':ab,ti,kw OR 'tests':ab,ti,kw)) OR generaliza*:ab,ti,kw OR generalisa*:ab,ti,kw OR 'concordance':ab,ti,kw OR ('intraclass':ab,ti,kw AND correlation*:ab,ti,kw) OR 'discriminative':ab,ti,kw OR 'known group':ab,ti,kw OR 'factor analysis':ab,ti,kw OR 'factor analyses':ab,ti,kw OR 'factor structure':ab,ti,kw OR 'factor structures':ab,ti,kw OR 'dimensionality':ab,ti,kw OR subscale*:ab,ti,kw OR 'multitrait scaling analysis':ab,ti,kw OR 'multitrait scaling analyses':ab,ti,kw OR 'item discriminant':ab,ti,kw OR 'interscale correlation':ab,ti,kw OR 'interscale correlations':ab,ti,kw OR ('error':ab,ti,kw OR 'errors':ab,ti,kw AND (measure*:ab,ti,kw OR correlat*:ab,ti,kw OR evaluat*:ab,ti,kw OR 'accuracy':ab,ti,kw OR 'accurate':ab,ti,kw OR 'precision':ab,ti,kw OR 'mean':ab,ti,kw)) OR 'individual variability':ab,ti,kw OR 'interval variability':ab,ti,kw OR 'rate variability':ab,ti,kw OR 'variability analysis':ab,ti,kw OR ('uncertainty':ab,ti,kw AND ('measurement':ab,ti,kw OR 'measuring':ab,ti,kw)) OR 'standard error of measurement':ab,ti,kw OR sensitiv*:ab,ti,kw OR responsive*:ab,ti,kw OR ('limit':ab,ti,kw AND 'detection':ab,ti,kw) OR 'minimal detectable concentration':ab,ti,kw OR interpretab*:ab,ti,kw OR (small*:ab,ti,kw AND ('real':ab,ti,kw OR 'detectable':ab,ti,kw) AND ('change':ab,ti,kw OR 'difference':ab,ti,kw)) OR 'meaningful change':ab,ti,kw OR 'minimal important change':ab,ti,kw OR 'minimal important difference':ab,ti,kw OR 'minimally important change':ab,ti,kw OR 'minimally important difference':ab,ti,kw OR 'minimal detectable change':ab,ti,kw OR 'minimal detectable difference':ab,ti,kw OR 'minimally detectable change':ab,ti,kw OR 'minimally detectable difference':ab,ti,kw OR 'minimal real change':ab,ti,kw OR 'minimal real difference':ab,ti,kw OR 'minimally real change':ab,ti,kw OR 'minimally real difference':ab,ti,kw OR 'ceiling effect':ab,ti,kw OR 'floor effect':ab,ti,kw OR 'item response model':ab,ti,kw OR 'irt':ab,ti,kw OR 'rasch':ab,ti,kw OR 'differential item functioning':ab,ti,kw OR 'dif':ab,ti,kw OR 'computer adaptive testing':ab,ti,kw OR 'item bank':ab,ti,kw OR 'cross-cultural equivalence':ab,ti,kw

AND

(('parent'/exp OR 'parent':ab,ti,kw OR 'proxy'/exp OR 'proxy':ab,ti,kw OR 'carer'/exp OR 'carer':ab,ti,kw OR 'caregiver'/exp OR 'caregiver':ab,ti,kw) AND ('report':ab,ti,kw OR 'reported':ab,ti,kw OR 'reporting':ab,ti,kw OR 'rated':ab,ti,kw OR 'rating':ab,ti,kw OR 'ratings':ab,ti,kw OR 'assessed':ab,ti,kw OR 'assessment':ab,ti,kw OR 'assessments':ab,ti,kw)) OR 'questionnaire'/exp OR 'questionnair*':ab,ti,kw OR 'instrument*':ab,ti,kw OR 'survey*':ab,ti,kw OR 'scal*':ab,ti,kw

NOT

<u>Potential 'NOT' terms</u>

NOT ('conference abstract'/it OR 'conference review'/it)
NOT ([animals]/lim NOT [humans]/lim)
NOT ('diseases'/exp/mj OR 'attention deficit disorder'/exp/mj OR 'autism'/exp/mj)

**SPORTDiscus**

<u>Population</u>

TI(infan* OR newborn* OR "new-born*" OR neonate* OR baby OR babies OR toddler* OR preschool* OR "pre-school*" OR kindergarten* OR childcare* OR daycare* OR nurser* OR ECEC OR "early childhood" OR "early years" OR "early life" OR children) OR AB(infan* OR newborn* OR "new-born*" OR neonate* OR baby OR babies OR toddler* OR preschool* OR "pre-school*" OR kindergarten* OR childcare* OR daycare* OR nurser* OR ECEC OR "early childhood" OR "early years" OR "early life" OR children) OR KW(infan* OR newborn* OR "new-born*" OR neonate* OR baby OR babies OR toddler* OR preschool* OR "pre-school*" OR kindergarten* OR childcare* OR daycare* OR nurser* OR ECEC OR "early childhood" OR "early years" OR "early life" OR children)

AND

<u>24h Movement behavior – Sleep</u>

SU(SLEEP OR "SLEEP-wake cycle" OR NAPS) OR TI(sleep* OR "time in bed" OR "bed time" OR bedtime OR "night rest" OR "night awakening*" OR "night waking*" OR "night wakening*" OR nap OR napping OR asleep OR AB(sleep* OR "time in bed" OR "bed time" OR bedtime OR "night rest" OR "night awakening*" OR "night waking* OR "night wakening*" OR nap OR napping OR asleep) OR KW(sleep* OR "time in bed" OR "bed time" OR bedtime OR "night rest" OR "night awakening*" OR "night waking*" OR "night wakening*" OR nap OR napping OR asleep)

AND

Measurement properties Proxy-report measures

SU(QUESTIONNAIR* OR SURVEY*) OR (DE ("CAREGIVERS") AND TI(report OR reported OR reporting OR rated OR rating OR ratings OR assessed OR assessment OR assessments)) OR (DE ("CAREGIVERS") AND AB(report OR reported OR reporting OR rated OR rating OR ratings OR assessed OR assessment OR assessments)) OR (DE ("CAREGIVERS") AND KW(report OR reported OR reporting OR rated OR rating OR ratings OR assessed OR assessment OR assessments)) OR TI(((parent OR proxy OR carer) AND (report OR reported OR reporting OR rated OR rating OR ratings OR assessed OR assessment OR assessments)) OR questionnair* OR instrument* OR survey* OR scal*) OR AB(((parent OR proxy OR carer) AND (report OR reported OR reporting OR rated OR rating OR ratings OR assessed OR assessment OR assessments)) OR questionnair* OR instrument* OR survey* OR scal*) OR KW(((parent OR proxy OR carer) AND (report OR reported OR reporting OR rated OR rating OR ratings OR assessed OR assessment OR assessments)) OR questionnair* OR instrument* OR survey* OR scal*)

NOT

Potential ‘NOT’ terms

(MM "CEREBRAL palsy" OR MM "ATTENTION-deficit hyperactivity disorder" OR MM "ATTENTION-deficit-disordered children" OR TI("cerebral palsy" OR autism OR autistic OR "attention deficit" OR ADHD) OR KW("cerebral palsy" OR autism OR autistic OR "attention deficit" OR ADHD))
