# Supplementary material for: A systematic review of proxy-report questionnaires assessing physical activity, sedentary behavior and/or sleep in young children (aged 0–5 years)
Source: Int J Behav Nutr Phys Act. 2022 Feb 14;19:18. doi: 10.1186/s12966-022-01251-x (PMC8845346; doi:10.1186/s12966-022-01251-x)
Supplement: Supplementary file 2 — Additional file 2. [file 12966_2022_1251_MOESM2_ESM.pdf]

Additional file 2: Methodological quality ratings of comparator instruments

Supplementary table 1: Methodological quality ratings of comparator instruments used for measuring physical activity, sedentary behavior and/or sleep in construct validity studies.

Measurement instrument	*Very good*	*Adequate*	*Doubtful*	*Inadequate*
**Accelerometer**	na	Accelerometer using cut-points/algorithm to classify level of activity, with at least moderate measurement properties in age group same as study sample and using the same accelerometer specifications (i.e., brand, epoch length, axis, device placement).<br><br>These ratings are based on the systematic review of Lettink et al.[1] (article in preparation).	Accelerometer using cut points/algorithm with some information on measurement properties in any age group.<br><br>These ratings are based on the systematic review of Lettink et al. [1] (article in preparation).	Accelerometer without providing information on measurement properties of cut-points/algorithm or evidence of insufficient measurement properties.
**Diary/questionnaire**	na	Diary/questionnaire with at least moderate measurement properties in age group same as study sample	Diary/questionnaire with some information on measurement properties in any age group	Diary/questionnaire without providing information on measurement properties or evidence of insufficient measurement properties
**Videosomnography (i.e., sleep behavior)**	Videosomnography with sufficient measurement properties in age group same as study sample	Videosomnography with moderate measurement properties in age group same as study sample	Videosomnography with some information om measurement properties in any age group	Videosomnography without providing information on measurement properties or evidence of insufficient measurement properties

Abbreviations: na not applicable

References

1. Lettink, A., et al., *Systematic review of accelerometer-based methods for 24-hour physical behavior assessment in young children (0-5-years-old)*. Article in preparation.
